# A critical analysis of computational protein design with sparse residue interaction graphs

**DOI:** 10.1371/journal.pcbi.1005346

**Published:** 2017-03-30

**Authors:** Swati Jain, Jonathan D. Jou, Ivelin S. Georgiev, Bruce R. Donald

**Affiliations:** 1 Computational Biology and Bioinformatics Program, Duke University, Durham, North Carolina, United States of America; 2 Department of Computer Science, Duke University, Durham, North Carolina, United States of America; 3 Department of Biochemistry, Duke University Medical Center, Durham, North Carolina, United States of America; 4 Department of Chemistry, Duke University, Durham, North Carolina, United States of America; Institute for Research in Biomedicine, SPAIN

## Abstract

Protein design algorithms enumerate a combinatorial number of candidate structures to compute the Global Minimum Energy Conformation (GMEC). To efficiently find the GMEC, protein design algorithms must methodically reduce the conformational search space. By applying distance and energy cutoffs, the protein system to be designed can thus be represented using a *sparse residue interaction graph*, where the number of interacting residue pairs is less than all pairs of mutable residues, and the corresponding GMEC is called the *sparse GMEC*. However, ignoring some pairwise residue interactions can lead to a change in the energy, conformation, or sequence of the sparse GMEC vs. the original or the *full GMEC*. Despite the widespread use of sparse residue interaction graphs in protein design, the above mentioned effects of their use have not been previously analyzed. To analyze the costs and benefits of designing with sparse residue interaction graphs, we computed the GMECs for 136 different protein design problems both with and without distance and energy cutoffs, and compared their energies, conformations, and sequences. Our analysis shows that the differences between the GMECs depend critically on whether or not the design includes core, boundary, or surface residues. Moreover, neglecting long-range interactions can alter local interactions and introduce large sequence differences, both of which can result in significant structural and functional changes. Designs on proteins with experimentally measured thermostability show it is beneficial to compute both the full and the sparse GMEC accurately and efficiently. To this end, we show that a provable, ensemble-based algorithm can efficiently compute both GMECs by enumerating a small number of conformations, usually fewer than 1000. This provides a novel way to combine sparse residue interaction graphs with provable, ensemble-based algorithms to reap the benefits of sparse residue interaction graphs while avoiding their potential inaccuracies.

## Author summary

Computational structure-based protein design algorithms have successfully redesigned proteins to fold and bind target substrates *in vitro*, and even *in vivo*. Because the complexity of a computational design increases dramatically with the number of mutable residues, many design algorithms employ cutoffs (distance or energy) to neglect some pairwise residue interactions, thereby reducing the effective search space and computational cost. However, the energies neglected by such cutoffs can add up, which may have nontrivial effects on the designed sequence and its function. To study the effects of using cutoffs on protein design, we computed the optimal sequence both with and without cutoffs, and showed that neglecting long-range interactions can significantly change the computed conformation and sequence. Designs on proteins with experimentally measured thermostability showed the benefits of computing the optimal sequences (and their conformations), both with and without cutoffs, efficiently and accurately. Therefore, we also showed that a provable, ensemble-based algorithm can efficiently compute the optimal conformation and sequence, both with and without applying cutoffs, by enumerating a small number of conformations, usually fewer than 1000. This provides a novel way to combine cutoffs with provable, ensemble-based algorithms to reap the computational efficiency of cutoffs while avoiding their potential inaccuracies.

## Introduction

Computational structure-based protein design is an emerging field with many applications in basic science and biomedical research [1]. Protein sequences have been designed to fold to specific tertiary structures [2–5]. Novel biological functions have been achieved by constructing new ligand binding sites and by switching binding specificities of enzymes [6–13]. New drugs, antibodies and nanobodies have been developed for therapeutic purposes by designing protein-protein and protein-ligand interfaces [14–19]. Negative design has also been used for predicting resistance mutations for highly drug-resistant pathogens [20, 21]. The above mentioned studies are examples of the predictive power of computational protein design algorithms.

A major challenge for protein design algorithms is to efficiently explore and evaluate a vast sequence and conformational search space, which increases exponentially with the number of design positions and mutations allowed. Most design algorithms use a rigid backbone and model side-chain flexibility using a discrete set of frequently-observed, low-energy conformations called *rotamers* [22]. However, even with a pairwise energy function, rigid backbone and rotamer libraries, identification of the Global Minimum Energy Conformation (GMEC) is NP-hard [23, 24]. Moreover, modeling of additional protein flexibility (both in the backbone and side chains) for realistic biological representation increases the search space [1–3, 6, 9, 10, 12, 25–33], which in turn results in increased runtime for protein design algorithms. Due to increased complexity, numerous heuristic techniques have been used to find a locally optimal solution and generate solutions quickly [6, 7, 25–27, 34–38]. Provable algorithms, on the other hand, guarantee the quality of the solutions found relative to the input model, and can generate a gap-free list of low-energy conformations within a given energy window of the GMEC [39]. One example is dead-end elimination (DEE) [28, 30–32, 40] followed by A* search [29, 41, 42], which has been used to approximate the thermodynamic ensemble and approximate the binding constant K_a_ [11, 43, 44]. However, in general provable algorithms require additional time and memory. With limited resources, it is important for design algorithms to systematically reduce the search space, without compromising on the quality and accuracy of design predictions. One way to do this is to use sparse residue interaction graphs for protein design, as described below.

### Sparse residue interaction graphs and protein design

Frequently, the goal of a protein design problem is to find the lowest energy sequences or conformations. Most protein design algorithms use pairwise energy functions to score protein conformations. Any such protein design problem can be represented by a *residue interaction graph*, where the nodes represent residues, and edges represent the interaction between residues. The energy functions usually consist of distance-dependent terms to model van der Waals and electrostatic interactions between residue pairs, and the interaction energy decreases with increasing distance between residues. Therefore, it is possible to neglect interaction energies between distant residues and not add them to the overall energy of a conformation. This eliminates edges between these negligibly-interacting residues from the residue interaction graph to construct a *sparse residue interaction graph*, with the corresponding GMEC called the *sparse GMEC*. We will refer to the residue interaction graph with no edges eliminated as the *full* residue interaction graph, and the corresponding GMEC as the *full GMEC*.

Whether explicitly described or not, the concept of sparse residue interaction graphs is ubiquitous in the field of protein design. Many design algorithms apply appropriate distance cutoffs implicitly in the energy function (using different cutoffs for different kinds of energies calculated) [24, 45–52], while others develop new algorithms to take explicit advantage of the sparseness of the residue interaction graph [53–60]. By using sparse residue interaction graphs, the number of interacting residue pairs is fewer than all pairs of mutable residues, and this reduces the effective search space considerably. However, the energies omitted by deleting the edges between negligibly interacting residues can add up, causing differences in sequence (such as amino acid identity) of the GMEC returned or the rankings of the top sequences (specific examples are discussed in detail in the Sections entitled “Results” and “Discussion”). While small sequence differences might not be consequential, larger differences in energies and sequences can lead to design algorithms returning a protein sequence that may not have the desired function. Therefore there is a potential tradeoff between reducing the search space and guaranteeing the accuracy and quality of the computed GMEC. Despite the widespread use of sparse residue interaction graphs in protein design, the effects of this tradeoff have not been previously analyzed.

In this paper, we present the results of our analysis of using sparse residue interaction graphs in protein design. We implemented a variation of the A* search algorithm in the protein design software osprey [43], for design with sparse residue interaction graphs to return the corresponding GMEC. osprey has been used in many successful designs *in vitro* [11, 13–15, 17, 18, 20], and even *in vivo* [14, 15, 18, 20]. We ran computational experiments on a total of 136 protein design problems, involving core, boundary, and surface residues. We used different energy and distance cutoffs to generate the sparse residue interaction graph, and analyzed the sequence and energy differences between the different GMECs returned. Our results show that commonly used distance cutoffs can return a GMEC whose sequence is different than that of the GMEC returned without those cutoffs. The underlying assumption when using distance and energy cutoffs is that neglecting long-range interactions do not have an effect on local interactions. We show that, contrary to this assumption, neglecting long-range interactions can alter favorable local interactions. Changes to the sequence and loss of favorable interactions between residues can both result in structural and functional changes to the predicted protein.

Next, in order to study if the sequence differences between the full and the sparse GMEC lead to functional differences, we performed retrospective validation on 6 protein design problems for which experimentally determined thermal stability data was available, and analyzed the sequences differences between the GMECs returned with and without distance cutoffs. Our analysis shows that across all 6 design problems, the sparse and full GMEC predicted different amino acid identities at 13 residues. Out of these 13 residues that have a different amino acid identity in the two GMECs, the more thermostabilizing mutation is predicted by the GMEC of the sparse residue interaction graph for 7 residues, and by the GMEC of the full residue interaction graph (without using distance cutoffs) for the remaining 6 residues. This indicates that there is no clear trend on which of the two GMECs will predict mutations with the desired function *in vitro*. Moreover, it can be difficult to correctly choose between the GMEC of the full residue interaction graph and its less computationally expensive sparse equivalent. Therefore it is beneficial to compute the GMECs for *both* the full and the sparse residue interaction graph, and to do so efficiently, while still taking advantage of the computational benefits of the reduced search space induced by the sparse residue interaction graph.

To achieve this goal, we provide a novel approach, called *Energy-bounding enumeration*, to combine sparse residue interaction graphs with provable, ensemble-based algorithms to generate both the GMECs efficiently. The gap-free list of low-energy conformations returned by an ensemble-based provable algorithm is guaranteed to contain the GMEC for the full residue interaction graph [59]. From this list, we prove that this GMEC can be found in additional *O*(*kn*^2^) time, where *n* is the number of mutable residues, and *k* is the number of conformations generated. We show that in practice, the full GMEC is almost always found within the first 1000 conformations returned. Because the number of conformations required to capture the GMEC is usually small, protein designers can henceforth combine sparse residue interaction graphs with provable, ensemble-based algorithms to exploit the reduced search space and still compute the GMECs for both the full and the sparse residue interaction graph. In short: sparse residue interaction graphs induce substantial differences in predicted sequences, conformations, and energies, with no way of telling which model will best predict the desired function. But provable, ensemble-based algorithms rescue computational protein design from these difficulties by providing a way to compute both GMECs efficiently.

In particular, this paper makes the following contributions:

1. Implementation of a variation of the A* search algorithm in the open-source protein design package osprey [11, 15–18, 20, 43] for protein design with sparse residue interaction graphs, and proof of the asymptotic time complexity to enumerate the GMEC from the gap-free list of conformations enumerated by this variant of A*.
2. Results showing that commonly used distance cutoffs can introduce large energy, conformation, and even sequence changes in the GMEC.
3. Examples showing that neglecting long-range interactions can alter local interactions, and an analysis of the sequence changes introduced when using sparse vs. full residue interaction graphs.
4. Retrospective designs and analysis of 6 protein design problems with experimentally measured thermostability drawn from the literature, emphasizing the benefits of computing the GMEC of both the full and the sparse residue interaction graph efficiently.
5. A novel approach, called *Energy-bounding enumeration*, to compute the GMEC of both the full and the sparse residue interaction graph in the gap-free list of conformations enumerated using the sparse residue interaction graph, and showing that the GMEC of the full residue interaction graph is usually found within the first 1000 conformations returned.
6. Examples of protein design problems in which A* with the full interaction graph failed to compute the GMEC, but using sparse residue interaction graphs allowed A* to compute the corresponding GMEC and also enumerate a gap-free list of conformations which is likely to contain the GMEC of the full graph.

## Materials and methods

### Definitions related to sparse residue interaction graphs

Each protein design problem is defined by its *input model*, namely, the input protein structure, the mutable residues, the allowed amino acids at each mutable residue, allowed side-chain conformations, and energy function.

Given this input model, the interaction energy between the mutable residues can be represented as an undirected graph, where vertices represent mutable residues, and edges represent pairwise interactions. An edge is present between two vertices when the pairwise energies between the two corresponding residues are included in the energy function. When the input energy function models all interactions between all mutable residues as pairwise energies, the *residue interaction graph* representing these interactions is the complete graph. We will refer to the complete residue interaction graph as the *full graph*. Every edge in the graph corresponds to a pairwise interaction between two mutable residues. By applying distance or energy cutoffs, the pairwise interactions between some mutable residues are omitted from the energy function. For every pair of residues (vertices) whose interactions are omitted, the corresponding edge between that pair is deleted from the residue interaction graph. This *sparse graph*, whose omitted edges correspond to the pairwise interactions omitted by the energy function, is called the *sparse residue interaction graph*. We will refer to the GMEC (which encodes both the conformation and sequence) of the full graph as the *full GMEC* and the GMEC of the sparse graph as the *sparse GMEC*. We will show that the full GMEC and sparse GMEC can be different, in both conformation and sequence. For any given conformation, we will refer to its computed energy with respect to the full graph as its *full energy*, and its energy with respect to the sparse graph as its *sparse energy*. For convenience, we will use *δ* and *α* to refer to distance and energy cutoffs, respectively:

- Distance cutoffs prune edges between two mutable residues whose minimum distance between any two atoms over all allowed rotamers is greater than a user-specified Euclidean distance *δ*.
- Energy cutoffs prune edges between two mutable residues whose maximum absolute pairwise energy over all allowed rotamers is less than a user-specified energy *α*.

**Fig 1:**
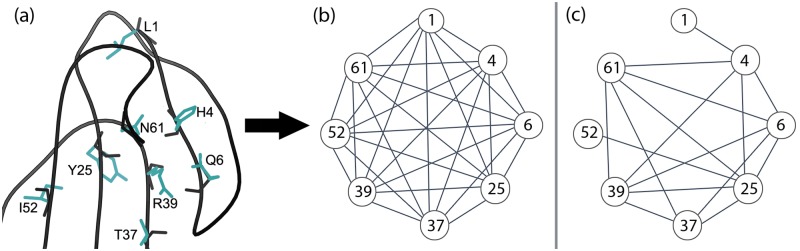
Example of a sparse residue interaction graph. (a) Cobrotoxin protein (PDB id: 1V6P) with the wild-type side chains of the 8 core mutable residues shown in cyan. (b) Design problem in (a) represented as a full residue interaction graph where all pairs of residues interact. (c) Design problem in (a) represented as a sparse residue interaction graph using a distance cutoff of *δ* = 8 Å.

Fig 1 shows the full and sparse residue interaction graphs for a protein design problem with 8 mutable residues. In this section, we have given high-level intuition for sparse residue interaction graphs, and their corresponding energy functions. For the proofs of Lemma 1 and Lemma 2 in S1 Text, however, precise definitions are useful to provide a mathematical basis for our claims. Hence, in S2 Text, we provide formal definition of a residue interaction graph, the sparse residue interaction graph, and the corresponding GMECs computed using such interaction graphs. We also provide a mathematical model for the cutoff criteria used to prune pairwise interactions from a residue interaction graph.

### Computational experiments

To study the sequence differences between the full and the sparse GMEC caused due to neglecting some pairwise energies, we need to compute the sparse GMEC first. We use the protein redesign package developed by the Donald lab, osprey for this study [43]. This section describes the changes made to osprey to compute the sparse GMEC, and the computational experiments designed to study the differences between the full and the sparse GMEC.

osprey uses dead-end elimination (DEE) followed by the A* search algorithm [41, 42, 61, 62] to provably return the GMEC and enumerate conformations in order of increasing energy. DEE prunes a significant portion of the possible conformations, and following that, A* search explores the unpruned search space to ensure that the first conformation returned is the GMEC. After the GMEC is returned, A* continues to enumerate conformations in increasing order of energy, until either all conformations within an energy window *E*_*w*_ of the GMEC are enumerated, or the number of conformations returned reaches a user defined number. A* guarantees that all conformations within *E*_*w*_ of the GMEC are returned, and the list of conformations enumerated is gap-free.

To generate the sparse GMEC, we modified the A* search algorithm used by osprey to calculate the sparse energy of a conformation (Eq. 2 in S2 Text). We will refer to this variant of the A* search algorithm as Sparse A*. Sparse A* evaluates conformations based on sparse energy (as opposed to full energy in traditional A* search), and can now be used for protein design with sparse residue interaction graphs. As Sparse A* retains all the guarantees provided by the A* search algorithm (because the algorithm is unmodified), the first conformation returned by Sparse A* is guaranteed to be the sparse GMEC, and it can also return a gap-free list of conformations within *E*_*w*_ of the sparse GMEC in increasing order of sparse energy. This property of Sparse A* is used to prove a surprising result, namely, that Sparse A* can efficiently generate not only the sparse GMEC, but also the full GMEC. This will be discussed later in the Section entitled “Discussion”.

Computational experiments were performed on the following design problems to generate the full and the sparse GMEC, and subsequently the energy, conformational, and sequence differences were analyzed:

- **Core designs**: 62 protein design problems, with the number of mutable residues ranging from 4-15 (each residue allowed to mutate to 5-10 amino acids) were taken from [32] and used with the same mutable residues and allowed amino acids.
- **Boundary designs**: PDB files for protein structures used in [32] were run through Naccess [63] to calculate the relative accessible surface area (RSA) for each residue. The residues with RSA between 20-50% were classified as boundary residues. Terminal residues, residues forming disulfide bonds, and prolines were not designed. 46 design problems were chosen and at most 20 residues were designed in each case. The residues were allowed to mutate to their wild-type identities and all amino acids except proline.
- **Surface designs**: PDB files for protein structures used in [32] were run through Naccess [63] to calculate the relative accessible surface area (RSA) for each residue. The residues with RSA between 50-80% were classified as surface residues. Terminal residues, residues forming disulfide bonds, and prolines were not designed. 28 design problems were chosen and at most 20 residues were designed in each case. The residues were allowed to mutate to their wild-type identities and all polar and charged amino acids.

In all experiments, the DEE pruning stage was followed by either A* to get the full GMEC, or the following two steps to generate the sparse GMEC and gap-free list of conformations: 1) sparse residue interaction graph generation using a user-defined distance cutoff *δ* or energy cutoff *α*, and 2) Sparse A* run to generate the sparse GMEC. For each design problem, Sparse A* was run four times using the following distance or energy cutoffs:

- *δ* = 8 Å;
- *δ* = 7 Å;
- *α* = 0.1 kcal/mol;
- *α* = 0.2 kcal/mol.

To further investigate how the differences in predicted sequence and conformation between the full and the sparse GMEC correlate with experimental measurements, we performed retrospective validation against 6 full-sequence designs from the literature, for which the designed mutants were experimentally determined to have improved thermal stability over the wild type [64–67]. Each example taken from the literature consisted of a protein redesign with an input structure together with experimentally measured melting point measurements showing a more thermostable designed mutant compared to the wild type sequence. We then performed computational redesign on the input structure. Consecutive residues of one or more adjacent secondary structures were allowed to either retain the wild-type identity or mutate to the amino acid identity of the designed, thermostabilized mutant. To compute the full GMEC, DEE pruning was followed by A* search. To compute the sparse GMEC, DEE pruning was followed by the generation of the sparse residue interaction graph with distance cutoff 7 Å, and then Sparse A* search. The number of mutable residues varied from 10-19 residues. The full and the sparse GMEC were then correlated against the measured melting point data.

The input model consists of a rigid backbone, rigid, discrete side-chain rotamers, and a pairwise energy function. All designs were done keeping the backbone fixed and modeling side-chain flexibility using the modal values of rotamers from the Penultimate rotamer library [22]. The energy function consisted of the amber van der Waals and electrostatic terms and the EEF1 pairwise implicit solvation model, as described in [11, 43]. Protein design formulations that consider additional side-chain flexibility [29, 32], backbone flexibility [28, 30, 31], free energy calculations [44, 61], or more accurate energy functions [68] have been developed. Nevertheless most of them call as a subroutine the simplified model discussed in this paper, which can be viewed as a core calculation common to most protein design software. Hence, the accuracy of this computation bounds the accuracy of the overall design. For more details on the input model, such as protein structures, mutable residues, and design protocol, please refer to S3 Text.

## Results

**Fig 2:**
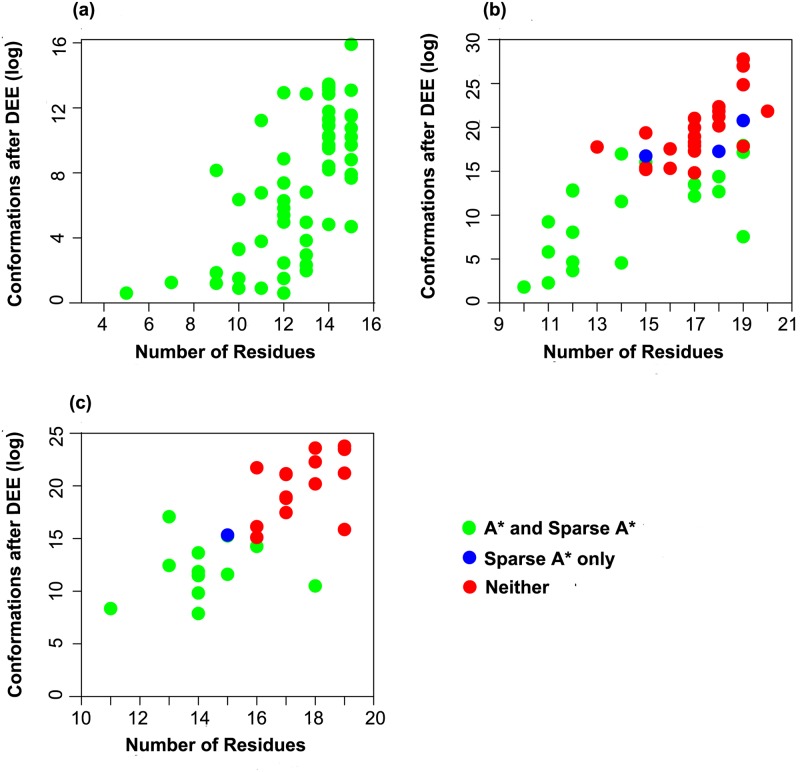
Overview of the 136 protein design test problems on 62 proteins studied in this paper. **Different problems required different amounts of resources**. (a) 62 core protein design problems, (b) 46 boundary design problems, and (c) 28 surface design problems. Design problems where A* returned the full GMEC and Sparse A* returned the sparse and the full GMEC are shown in green. Design problems where A* ran out of memory (30GB) before returning the full GMEC and Sparse A* returned the sparse GMEC are shown in blue. Design problems where both A* and Sparse A* ran out of memory (30GB) before returning any conformation are shown in red.

Out of the 62 core, 46 boundary, and 28 surface protein redesign problems, A* returned the full GMEC, and Sparse A* returned the sparse GMEC (with all four distance and energy cutoffs given in the Section entitled “Computational experiments“) for all 62 core, 21 boundary, and 12 surface design problems, shown as green dots in Fig 2. These design problems were used to study the effects of different distance and energy cutoffs. Three kinds of differences were studied between the full and the sparse GMEC for each of the distance and energy cutoffs: rotamer, sequence, and energy. The following subsections (entitled “Core redesign”, “Boundary redesign”, and “Surface redesign”) discuss the details of this analysis. The protein design problems for which only Sparse A* run finished (blue points) and for which both A* and Sparse A* ran out of memory (red points) are discussed in the Section entitled “Discussion”.

### Core redesign

For 62 core designs, Sparse A* with both distance cutoffs returned the sparse GMEC identical to the full GMEC for all 62 design problems, and in 59 design problems with energy cutoff *α* = 0.1 kcal/mol, and in 58 design problems with energy cutoff *α* = 0.2 kcal/mol. For the four core problems where the full GMEC was different from the sparse GMEC, the full GMEC was the second conformation returned by Sparse A*, and the energy difference between the full and the sparse GMEC was less than the energy cutoff of 0.2 kcal/mol. Out of these four design problems, two had sequence differences between the full and the sparse GMEC: the human sulfite oxidase cytochrome b5 domain (PDB id: 1MJ4) and bacterial iron-sulfur protein (PDB id: 3A38) had single amino acid differences between the full and the sparse GMEC (residue 50 for 1MJ4 and residue 26 for 3A38). In both cases, serine in the full GMEC was replaced by alanine in the sparse GMEC. Except for these two cases, distance and energy cutoffs did not have sequence-changing effects on the GMEC returned for core designs. Interestingly, using an energy cutoff of 0.2 kcal/mol results in omitting a large fraction of residue pairs for most of the core design problems, between 45% to 80% (S1 Fig). Despite the tightly packed nature of the protein core, the energy interactions were less than 0.2 kcal/mol, which is less than the typical van der Waals interaction energy of 0.5-1 kcal/mol.

### Boundary redesign

**Fig 3:**
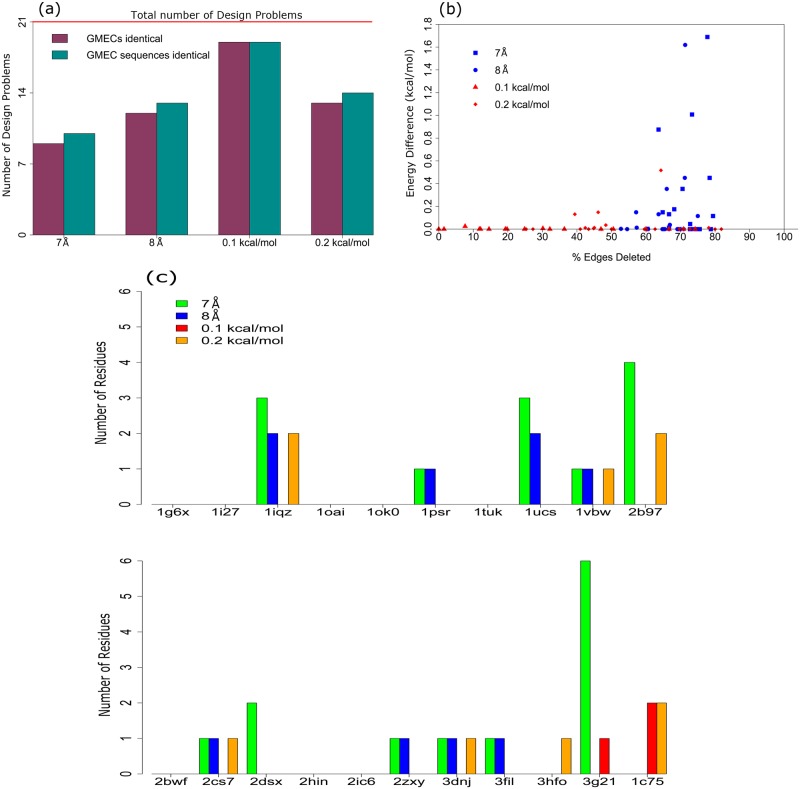
Sparse residue interaction graphs introduce differences in energy, conformation, and sequence of the GMEC. Data shown for 21 boundary design problems, for each of which Sparse A* was run with the following cutoffs: distance cutoff *δ* = 8 Å, *δ* = 7 Å, energy cutoff *α* = 0.1 kcal/mol and *α* = 0.2 kcal/mol. Number of mutable residues in each design problem ranged from 10-20. (a) Number of design problems where full GMEC and sparse GMEC are identical (purple), and where the sequences of the full GMEC and sparse GMEC are identical (cyan). The total number of boundary design problems (21) is indicated by the horizontal red line. (b) Percentage of edges deleted from the residue interaction graph vs. the full energy difference between full GMEC and sparse GMEC. (c) Number of residues with different amino acids between the full GMEC and the sparse GMEC. *y*-axis value of 0 indicates that the sequences of the full GMEC and the sparse GMEC are identical.

Unlike core designs, the number of boundary design problems where the sparse GMEC was identical to the full GMEC was larger for energy cutoffs than distance cutoffs, as shown in Fig 3(a). The energy cutoff of *α* = 0.1 kcal/mol gave the best results, returning the sparse GMEC identical to the full GMEC in 19 out of the 21 boundary design problems. For problems where the full GMEC and the sparse GMEC were different, the full energy and sequence difference between the full and the sparse GMEC are larger for distance cutoffs than for energy cutoffs, (Fig 3(b) and 3(c)), with the distance cutoffs introducing sequence differences in a total of 24 residues as compared to 11 residues with energy cutoffs (S1 Table). For a single design problem, this number can be as high as 6 (C-terminal domain of the Rous Sarcoma Virus capsid protein, PDB id: 3G21), which is more than one-third of the 15 mutable residues for that design problem.

### Surface redesign

**Fig 4:**
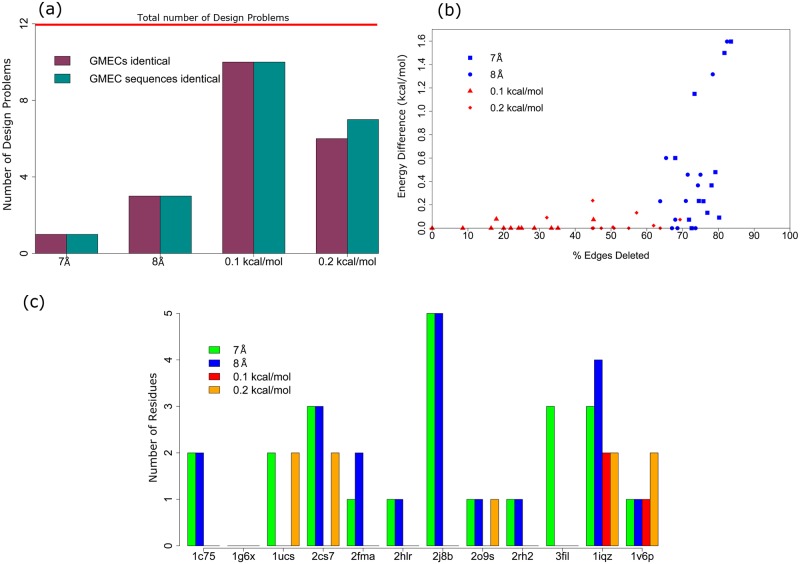
Sparse residue interaction graphs introduce differences in energy, conformation, and sequence of the GMEC. Data shown for 12 surface design problems, for each of which Sparse A* was run with the following cutoffs: distance cutoff *δ* = 8 Å, *δ* = 7 Å, energy cutoff *α* = 0.1 kcal/mol and *α* = 0.2 kcal/mol. Number of mutable residues in each design problem ranged from 11-19. (a) Number of design problems where full GMEC and sparse GMEC are identical (purple), and where the sequences of the full GMEC and sparse GMEC are identical (cyan). The total number of boundary design problems (12) is indicated by the horizontal red line. (b) Percentage of edges deleted from the residue interaction graph vs. the full energy difference between full GMEC and sparse GMEC. (c) Number of residues with different amino acids between the full GMEC and the sparse GMEC. *y*-axis value of 0 indicates that the sequences of the full GMEC and the sparse GMEC are identical.

Similar to boundary design problems, energy cutoffs returned a sparse GMEC which was identical to the full GMEC in more cases than distance cutoffs did. The sparse GMEC is identical to the full GMEC in 10 out of the 12 surface design problems for energy cutoff *α* = 0.1 kcal/mol, and only for 1 out of 12 for distance cutoff *δ* = 7 Å (Fig 4(a)). Sequence differences between the full and the sparse GMEC occur even though the energy differences between these GMECs are small. Unlike boundary designs, where in a few cases the energy cutoffs introduced more sequence differences between the full and the sparse GMEC, the energy cutoff of *α* = 0.1 kcal/mol has a smaller or equal number of sequence differences than distance cutoffs in all 12 surface design problems, as shown in Fig 4(c). Overall, distance cutoffs introduced sequence differences in a total of 25 residues, as compared to 9 residues for energy cutoffs (S2 Table).

Since distances between residues on the surface of the protein are larger as compared to boundary or core regions, Sparse A* was run for the 12 surface designs problems again with a distance cutoff *δ* = 10 Å. This resulted in the sparse GMEC being identical to the full GMEC for 5 out of 12 design problems, as compared to 3 out of the 12 design problems for *δ* = 8 Å. For one additional case (bacterial oxidized ferredoxin protein, PDB id: 1IQZ), the number of amino acid sequence differences was reduced. However, this still only increases the number of design problems for which the full GMEC and the sparse GMEC are identical to half of the 10 when using the energy cutoff of *α* = 0.1 kcal/mol. Overall, increasing the distance cutoff from 8 Å to 10 Å decreased amino acid differences in only 3 out of the 9 design problems. In human CD59 glycoprotein (PDB id: 2J8B), the number of amino acid sequence differences is 5 with distance cutoffs of 7 Å, 8 Å, and 10 Å, and increasing the distance cutoff led to no change in the sequence difference between the full and the sparse GMEC whatsoever.

### Large long-range interactions neglected by distance cutoffs

**Fig 5:**
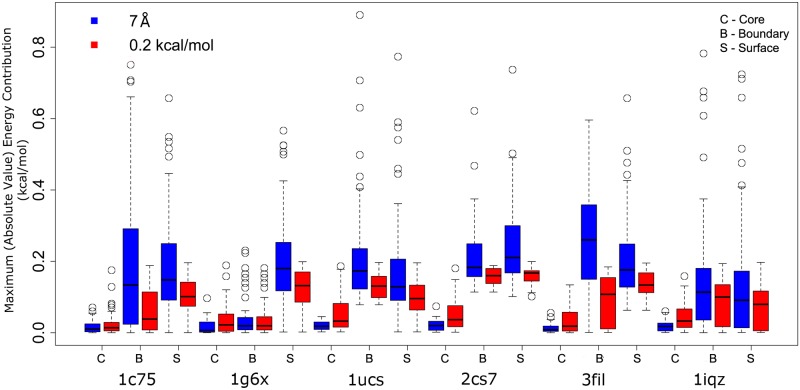
Distance cutoffs can delete edges with large (almost 0.9 kcal/mol per edge) interaction energy. The maximum (in absolute value) value of interaction energy for 18 test cases: each deleted edge with distance cutoff *δ* = 7 Å (blue) and energy cutoff *α* = 0.2 kcal/mol (red), for core (C), boundary (B), and surface (S) designs for 6 protein structures.

The above results suggest that using distance cutoffs can neglect long range interactions between residue pairs, and cause significant sequence differences between the GMECs returned (S2 Fig, S1 and S2 Tables). Using a larger distance cutoff (10 Å) did little to improve the results. Neglecting these long range interactions tends to have a larger effect on boundary and surface designs than on core designs. To investigate this further, the maximum energy (in absolute value) contributed over all rotamer pairs by each of the edges deleted using distance and energy cutoffs were analyzed. To eliminate any uncertainties, the results of core, boundary, and surface designs on the same protein structure were used for this analysis. Fig 5 shows the distribution of omitted pairwise energies of sparse graphs generated with either distance or energy cutoffs for 36 different protein design problems over 6 different structures. For the 6 protein structures shown in Fig 5, using distance cutoffs in boundary and surface designs can delete edges from the residue interaction graph with larger interaction energies, as compared to using energy cutoffs. The opposite occurs for core designs. This is consistent with the fact that both the distance cutoff *δ* = 7 Å and energy cutoff *α* = 0.2 kcal/mol had similar results for core designs, but for boundary and surface designs, the number of residues with different amino acids between the full and the sparse GMEC is larger for the distance cutoff than for the energy cutoff, except for bacterial cytochrome C-553 protein (PDB id: 1C75). By definition, energy cutoffs only delete an edge when its maximum absolute energy contribution is smaller than the specified limit. By contrast, distance cutoffs delete edges whose energy contributions can vary arbitrarily from being very small (0.05 kcal/mol) to being very large (almost 0.9 kcal/mol). As such, our results foreground a key difference between distance cutoffs and energy cutoffs: in terms of the energy contributed by each edge, precomputed energy cutoffs are more precise. While distance cutoffs omit any sufficiently distant pairwise interaction, providing limited control over the energy contributions of the omitted edges, energy cutoffs will never omit any pairwise interaction that can exceed the specified energy cutoff. Therefore energy cutoffs allow greater precision in selection of low-energy pairwise interactions.

**Fig 6:**
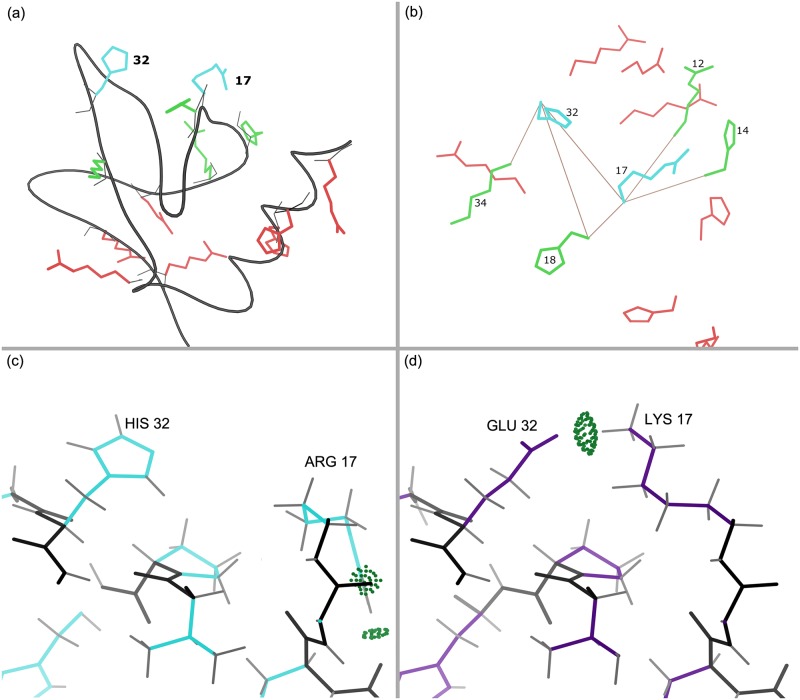
Sequence differences with full vs. sparse residue interaction graphs: hydrogen bond is disrupted when long-range interactions are omitted. Comparison between the sequences of the full and sparse GMEC for the surface design of domain of pneumococcal histidine triad A protein (PDB id: 2CS7) are shown. (a) Mutable residues of the sparse GMEC. Protein backbone is shown in black. Residues 17 and 32 are shown in cyan. With distance cutoff *δ* = 8 Å, the interactions between red and cyan residues are eliminated in the sparse residue interaction graph. (b) Solid brown lines indicate residues interacting with the cyan residues in the sparse residue interaction graph. Amino acids at residues 17 and 32 from the sparse GMEC are shown in cyan. (c) Residues 17 and 32 of the sparse GMEC. (d) Residues 17 and 32 of the full GMEC. Note that the hydrogen bond between residues 17 and 32 (Lys:Glu) in the full GMEC (d) is lost in the sparse GMEC (c), where the side chain of residue 17 (Arg) forms hydrogen bonds with nearby backbone atoms. Hydrogen bonds are shown as green dotted-pillows that indicate the overlap between the vdW spheres of the hydrogen and the acceptor atom, generated using Probe [69].

**Fig 7:**
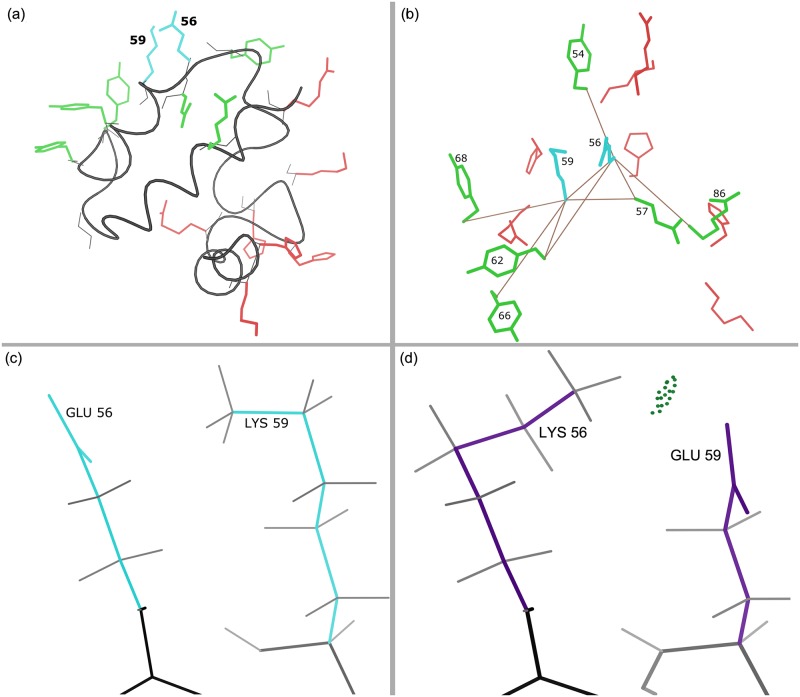
Sequence differences with full vs. sparse residue interaction graphs: hydrogen bond is disrupted when long-range interactions are omitted. Comparison between the sequences of the full and sparse GMEC for the surface design of bacterial cytochrome C-553 protein (PDB id: 1C75) are shown. (a) Mutable residues of the sparse GMEC. Protein backbone is shown in black. Residues 56 and 59 are shown in cyan. With distance cutoff *δ* = 8 Å, the interactions between red and cyan residues are eliminated in the sparse residue interaction graph. (b) Solid brown lines indicate residues interacting with the cyan residues in the sparse residue interaction graph. Amino acids at residues 56 and 59 from the sparse GMEC are shown in cyan. (c) Residues 56 and 59 of the sparse GMEC, where the hydrogen bond is lost. (d) Residues 56 and 59 of the full GMEC, that form the hydrogen bond. Hydrogen bonds are shown as green dotted-pillows that indicate the overlap between the vdW spheres of the hydrogen and the acceptor atom, generated using Probe [69]. This hydrogen bond is lost in the sparse GMEC (c).

One reason distance cutoffs are widely used is the assumption that sufficiently distant interactions do not affect local interactions. Our results indicate that this is not always true. Figs 6 and 7 show the sequence differences between the full GMEC and the sparse GMEC with distance cutoff *δ* = 8 Å in two such examples from bacterial cytochrome C-553 protein (PDB id: 1C75) and a domain of pneumococcal histidine triad A protein (PDB id: 2CS7). In both cases, neglecting the interaction between the distal residues (red) and the two proximal residues (cyan) leads to a missing hydrogen bond. In Fig 6, the amino acids of the full GMEC are replaced with entirely different amino acids. In the sparse GMEC, residue 17 is an arginine instead of a lysine, and residue 32 is a histidine instead of a glutamic acid. While residue 17 and residue 32 of the full GMEC form a hydrogen bond, in the sparse GMEC the arginine at residue 17 forms hydrogen bonds with the backbone instead. In Fig 7, the amino acids at residues 56 and 59 are swapped between the full and the sparse GMEC. These examples illustrate two cases in which neglecting long-range interactions can disrupt favorable local interactions. In general, for the design problems analyzed in this paper, the disruption of local interactions is more common in surface designs. For 4 surface design problems, the interaction between two mutable residues is different in the full and the sparse GMEC. In 3 of these cases a favorable local interaction is lost by using distance cutoff 7 Å.

### Retrospective validation against experimental data

To study how well the sequence differences between the full and the sparse GMEC correlate with experimental measurements, we conducted retrospective design experiments. As described in the methods section (entitled “Computational experiments”), we took examples of wild-type proteins from the literature which were computationally redesigned to improve thermostability (the designed protein had higher T_m_ than the wild-type protein) [64–67], and redesigned a subset of each protein, allowing mutable residues to either retain their wild-type identity or mutate to the corresponding amino acid identity of the more stable designed mutant. We computed the full and the sparse GMEC for all 6 design problems and then compared the GMECs to identify differences in sequence, energy, and conformation. We correlated the difference in sequence with experimentally measured melting temperature data. Amino acid identities were restricted in the redesign procedure to ensure that any difference in sequence between the full and the sparse GMEC would correspond to either the less or the more stable protein sequence, and hence could be directly validated against the melting temperature data. The designed search space ranged from 6.65 × 10^15^ to 6.13 × 10^25^ conformations (see the section entitled “Rank of full GMEC in practice”).

For 5 of the 6 protein design problems, amino acid differences between the full and sparse GMEC were found. In the sixth design problem, the sparse GMEC and full GMEC predicted the same sequence, but have different side-chain conformations. Table 1 lists the residue numbers for the 5 problems in which the full and sparse GMEC differed in sequence. For the two residues that have different amino-acid identity between the full and the sparse GMEC for the B1 domain of protein L (PDB id: 1HZ5), the full GMEC predicts the amino acid identities in the more stable designed protein for both Lys 4 and Glu 26. In contrast, for the U1 nuclear ribonucleoprotein A (PDB id: 1URN) the sparse GMEC predicts the amino acid identity in the more stable protein for both Gln 93 and Asp 97. For the designed engrailed homeodomain dimer (PDB id: 2MG4), the sparse GMEC predicts the amino acid identity of the more stable protein for both Arg 52 and Glu 54. For the remaining two protein design problems, at some residues the full GMEC predicts the amino acid identity of the more stable designed protein, and for other residues the sparse GMEC predicts the amino acid at the more stable designed protein instead. Comparison of the complete sequences of the sparse and full GMEC can be found in the S3 Table. In summary, for the 5 design problems, there are 13 residues where the sparse and full GMEC predicted different amino acids. For these 13 residues, the amino acid identity of the more stable designed protein is predicted by the full GMEC for 6 residues, and by the sparse GMEC for the other 7 residues. These results suggest that when using a rigid backbone, rigid rotamer, GMEC-only input model and sparse or full pairwise energy function, it is unclear which of the two GMECs (sparse or full) will correspond to the desired protein function (in this case, improved thermostability).

**Table 1:** Sequence correlation between designed mutant and wild type. The table shows mutable residues at which the sparse and full GMEC predict different amino acid identities: one predicted the amino acid identity of the more stable designed mutant, and the other predicted the amino acid identity of the less stable wild type. The amino acid identity of the designed mutant is in bold. The wild-type amino acid identity is not in bold.

Protein Structure	PDB id	Residue Number	Full GMEC^a^	Sparse GMEC^b^
Protein L [65]	1HZ5	4	**Lys**	Val
		26	**Glu**	Phe
U1A [65]	1URN	93	Ile	**Gln**
		97	Met	**Asp**
Engrailed Homeodomain of *D. melanogaster* [64]	1ENH	47	Ile	**Gln**
		55	**Arg**	Lys
Symmetric protein homodimer [66]	2MG4	52	Asn	**Arg**
		54	Arg	**Glu**
Acylphosphatase [65]	2ACY	8	Gln	**Ser**
		10	Lys	**Asp**
		14	**Lys**	Phe
		16	**Asp**	Lys
		76	**Lys**	Asp

^a^Full GMEC amino acid identity

^b^Sparse GMEC amino acid identity.

**Fig 8:**
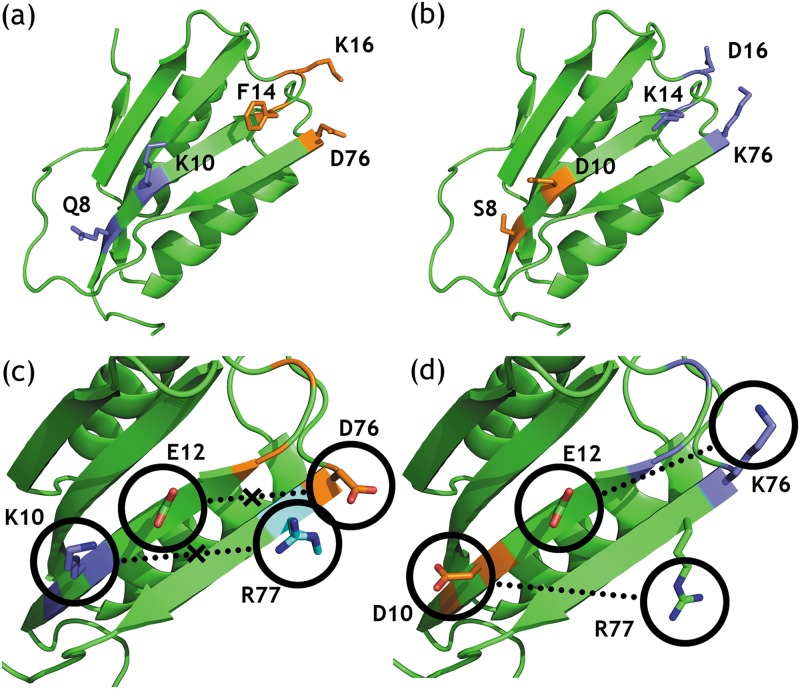
Sequence differences between full vs. sparse residue interaction graphs: biophysically significant long-range interactions are omitted and change the sequence of the GMEC. Comparison between the sequences of the full and sparse GMEC for the restrospective design of acyl phosphatase (PDB id: 2ACY) are shown. Protein backbone is shown as a green ribbon. (a) and (b) show the residues where the sparse and full GMEC have different amino acid identities: (a) shows the sparse GMEC, and (b) shows the full GMEC. Residues for which the amino acid identity is that of the thermostabilized mutant are shown in blue. Residues for which the amino acid identity is that of the wild-type are shown in orange. (c) and (d) show the omitted pairwise interactions in the sparse GMEC and their corresponding interactions in the full GMEC. Residues whose sequence and conformation are that of the full GMEC are shown in green. Arg 77 has a different side-chain conformation in the sparse GMEC, and is highlighted in cyan. Oxygen and nitrogen atoms are colored to show favorable and unfavorable interactions. (c) With distance cutoff *δ* = 7 Å, the unfavorable interactions between Glu 12 and Asp 76 and Lys 10 and Arg 77 are omitted in the sparse residue interaction graph, and not considered when computing the sparse GMEC. (d) In the full GMEC these interactions are replaced with favorable interactions instead.

We then analyzed the sparse residue interaction graph to determine the significance of the omitted edges. In particular, we identified pairwise interactions whose omission would change the sequence of the computed GMEC. Table 2 lists these omitted pairwise interactions, the minimum distance (closest Euclidean inter-residue distance between any two atoms when all rotamer combinations for the two residues are considered, see the Section entitled “Sparse residue interaction graphs and protein design”) between the interacting residue pair, the total difference in energy between the full and sparse GMEC, and the difference in energy contributed by these omitted edges to the sparse and full GMEC. The omission of the pairwise interactions listed in Table 2 alone was large enough to change the sequence of the computed GMEC, and even lead to changes in experimental measurements. For example, in the case of acyl phosphatase (PDB id: 2ACY), Fig 8 shows the sequence differences and key high-energy long-range interactions omitted in the sparse residue interaction graph. In the full GMEC the minimum distance between residues Glu 12 and Lys 76 is 7.6 Å, and its high-energy long-range interaction of -0.34 kcal/mol is omitted in the sparse residue interaction graph. The minimum distance between residues Asp 10 and Arg 77 is 8.6 Å, and its high-energy long-range interaction of -0.26 kcal/mol is omitted in the sparse residue interaction graph. The corresponding pairwise energy between residues Glu 12 and Asp 76 in the sparse GMEC is 0.318 kcal/mol, and the corresponding pairwise energy between residues Glu 12 and Asp 76 in the sparse GMEC is 0.314 kcal/mol. This amounts to a total difference of 1.24 kcal/mol. The energies of the sparse and full GMEC differ by only 0.75 kcal/mol. As can be seen in panel (c) of Fig 8, the sparse GMEC neglects the energetically unfavorable interactions between both the negatively charged glutamic acid at residue 12 and aspartic acid at residue 76, and the positively charged lysine at residue 10 and arginine at residue 77. These unfavorable long-range electrostatics are not found in the full GMEC, as seen in panel (d). Omitting these two pairwise interactions alone would change the sequence of the corresponding GMEC. Note that in this design problem, large, favorable electrostatic pairwise interactions in the full GMEC are replaced with large, unfavorable electrostatic interactions in the sparse GMEC. The cumulative difference is greater than 1.2 kcal/mol, which is large enough to be biophysically relevant.

**Table 2:** Omitting key high-energy long-range interactions changes the sequence of the GMEC. The table shows the one or two highest energy interactions omitted by a distance cutoff of 7 Å. The omission of these edges alone is sufficient to change the sequence of the GMEC computed using a sparse residue interaction graph.

PDB id	High-energy Pairs^a^	Distance^b^(Å)	Full Energy Difference^c^ (kcal/mol)	Omitted Energy Difference^d^ (kcal/mol)
1HZ5	(23, 26)	7.1	0.16	0.393
1URN	(90, 97)	7.4	0.175	0.462
1ENH	(43, 52), (44, 52)	10.7, 7.86	0.181	0.231
2MG4	(8, 54)	14.3	0.201	0.356
2ACY	(10, 77), (12, 76)	8.6, 7.6	0.75	1.24
1VJQ	(12, 22), (18, 24)	9.92, 8.57	0.121	0.214

^a^Residue numbers of the two mutable residues in the omitted pairwise interaction

^b^Minimum distance *d*_*min*_(*i*, *j*) (see S2 Text) of the corresponding residue pair

^c^Energy difference between the sparse GMEC and full GMEC using the full energy function (Eq. 1 in S2 Text)

^d^Cumulative energy difference between the sparse and full GMEC for only the pairwise interactions *in column (a)*

Even when the sparse energy function predicts amino acid identities of the more thermostable designed mutant, the difference in energy between the sparse GMEC and full GMEC can manifest as a difference in backbone coordinates and side-chain conformations. We analyzed an example of this, where the full GMEC predicts a rotamer that closely resembles the wild type while the sparse GMEC predicts a rotamer which has a *χ*_1_ angle difference of 90.6°. For human procarboxypeptidase A2 (PDB id: 1AYE), the sequences of the full and sparse GMECs were identical, but the residue conformations differed. To test if sparse residue interaction graphs could be a source of conformational difference between the predicted and experimentally observed residue conformations, we performed side-chain placement using osprey on the designed mutant of human procarboxypeptidase A2 (PDB id: 1VJQ). The structure of the designed mutant was used as input to rule out backbone changes as an additional source of error. We analyzed the conformations of the sparse GMEC, the full GMEC, and the crystal structure, and found the predicted conformations of sparse and full GMEC differed at two residues. At residue Glu 18, the rotamer of the full GMEC coincides closely with the crystal structure, whereas the rotamer of the sparse GMEC had a 90.6° difference in its *χ*_1_ angle, differing significantly from crystal structure. At residue Lys 22, both the sparse and the full GMEC preserve the overall direction of the charged amine group, but their *χ*-angles differ from the crystal structure.

In the side-chain placement problem for the designed mutant of human procarboxypeptidase A2 (PDB id: 1VJQ), two pairwise interactions contributed the most to the energy difference between the sparse and full GMEC: the pairwise interactions between residues Glu 12 and Lys 22, and residues Glu 18 and Asp 24. The minimum distance between residues Glu 12 and Lys 22 is 9.9 Å, and in the full GMEC their long-range interaction of -0.42 kcal/mol is omitted in the sparse residue interaction graph. The corresponding pairwise energy between residues 12 and 22 in the sparse GMEC is -0.32 kcal/mol. The minimum distance between residues Glu 18 and Asp 24 is 8.6 Å, and in the full GMEC its long-range interaction of 0.21 kcal/mol is omitted in the sparse residue interaction graph. The corresponding pairwise energy between residues Glu 18 and Asp 24 in the sparse GMEC is 0.32 kcal/mol. The energy difference from these two edges account for a cumulative difference of 0.21 kcal/mol. The energies of the sparse and full GMEC differ by 0.16 kcal/mol. Omitting these two pairwise interactions alone would change the conformation of the computed GMEC.

## Discussion

Our results not only show how omitted pairwise interactions can change the computed sequence of the GMEC in computational protein design, but also that these differences in sequence can correlate with experimental measurements. In the examples where the full GMEC predicts mutations that correlate with the thermostable mutant but the sparse GMEC does not, key high-energy, long-range pairwise interactions are consistently omitted from the sparse residue interaction graph and this failure to account for long-range interactions changes the sequence of the sparse GMEC. Even in cases where the sparse GMEC better correlates with the designed mutant, the energy contribution of pairwise interactions that are omitted by distance cutoffs is significant.

In this study, we imposed many modeling assumptions: the backbone is rigid, side-chain flexibility is confined to rigid rotamers, and a single conformation, namely the GMEC, is assumed to be representative of the thermodynamic ensemble. Since the GMEC (sparse or full) is the provably optimal sequence and structure predicted by the input model, and since this model is commonly used as a subroutine in many empirical designs [1, 24, 39, 45–60], our results represent a bound on how well any algorithm can do, using either the sparse or full input models. Our results show that commonly used distance cutoffs, especially when computed without bounding the difference in energy between the sparse and full GMEC, do in fact introduce error into any computational structure-based protein design protocol. Although this error does not always negatively impact downstream experiments, the computational expedience derived from applying distance cutoffs can come with a cost: measurable loss in accuracy. To overcome this cost, algorithms that merely compute a low-energy locally optimal solution or even the GMEC of the sparse residue interaction graph are inadequate. Therefore, it will be highly beneficial if we can compute both the full and the sparse GMEC efficiently, while still harnessing the computational advantages of the reduced search space provided by sparse residue interaction graphs. In this section we describe how to efficiently compute both the full and the sparse GMEC, by a novel approach that combines sparse residue interaction graphs with ensemble-based design algorithms. The new approach, called *Energy-bounding enumeration*, is comparable in speed to sparse GMEC search on the sparse residue interaction graph.

### Full GMEC with Sparse A*

As described in the Section entitled “Definitions related to sparse residue interaction graphs”, sparse residue interaction graphs are generated by omitting distant or low-energy pairwise interactions from the energy function. These sparse graphs correspond to modified energy functions. Naturally, modifying the energy function can change the GMEC. The conformation and sequence of the GMEC of the full graph may be different from the GMEC of the sparse graph. However, for any pair of mutable residues, the maximum and minimum contributions of their pairwise interactions bound their total contribution to any conformation. Furthermore, their maximum and minimum contributions are efficient to compute. Thus, the contribution of any pairwise interaction can be efficiently bounded, and the cumulative maximal and minimal contribution of all omitted pairwise interactions can be efficiently bounded as well. These cumulative maxima and minima can be mathematically combined to bound the energy difference between the sparse and full GMEC (Lemma 1, S1 Text). Although the bounds given by Lemma 1 are often loose, it is important to know that the energy difference between the full and the sparse GMEC can always be bounded. This energy bound guarantees that a gap-free list (enumerated by a provable algorithm such as Sparse A*), that contains the sparse GMEC and all conformations within that energy bound of the sparse GMEC, will contain both the sparse and full GMEC. If we simply compute the full energies of all conformations in this list, then the conformation with the lowest full energy is guaranteed to be the full GMEC. Furthermore, computing the full energies for all conformations in the list is efficient (Lemma 2, S1 Text).

The fact that Sparse A* can enumerate both the sparse and the full GMEC means that we no longer have to worry about which of the two sequences (the full or the sparse GMEC) will predict the desired functional mutations. One can generate the sparse residue interaction graph, calculate the upper bound on the energy difference, and run Sparse A* to return a gap-free list of conformations that is guaranteed to return the full GMEC. The list of conformations returned by Sparse A* can then be re-ranked based on the full energy to get the full GMEC. Once both the full and the sparse GMEC are found, they can be evaluated based on more sophisticated methods for energy calculation [68, 70], or any other method that seems pertinent to the designer. Note that both the full and the sparse GMEC are guaranteed to be found only when using provable ensemble-based algorithms that are guaranteed not to miss any conformation within the specified energy window. The re-ranking can be done relatively quickly (Lemma 2 in S1 Text), when the number of conformations that need to be generated by Sparse A* to find the full GMEC is not large. Both the full and the sparse GMEC were found for most of the design problems used in this study, and this is discussed in the Section entitled “Rank of full GMEC in practice”.

In this section, we have provided high-level intuition showing how Sparse A* can be used to compute both the sparse and full GMEC. These statements are supported by mathematical guarantees, which show that the full GMEC is contained in the gap-free list enumerated by Sparse A*, and that it is efficient to compute the full GMEC from that list. In S1 Text we provide two Lemmas and their proofs. Lemma 1 proves an upper bound on the absolute difference in sparse energy between the sparse and full GMEC, and Lemma 2 gives the time complexity to compute the full GMEC from a gap-free list guaranteed to contain the full GMEC. We then describe how these two proofs are sufficient to compute both the sparse and full GMEC using Sparse A*. Finally, we briefly describe a recent provable algorithm [59], which uses concepts from dynamic programming to exploit the optimal substructure induced by sparse graphs, and achieve asymptotic time complexity significantly better than the worst-case time complexity of any algorithm using the full graph.

### Rank of full GMEC in practice

**Fig 9:**
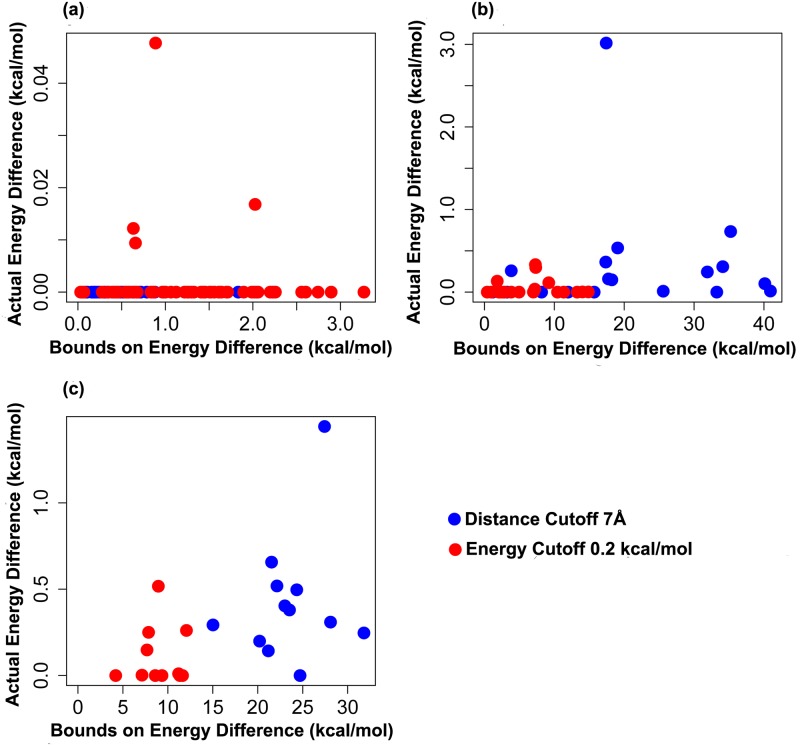
Actual sparse energy difference between the full and sparse GMEC is much smaller than the theoretical energy bound. Bounds on the sparse energy difference (as calculated by Lemma 1 in S1 Text) vs. the actual full energy difference between the full GMEC and sparse GMEC for distance cutoff *δ* = 7 Å (blue) and energy cutoff *α* = 0.2 kcal/mol (red). (a) 62 core protein design problems, (b) 21 boundary protein design problems, (c) 12 surface protein design problems.

Fig 9 plots the calculated upper bounds vs. the actual full energy difference between the full and the sparse GMEC for the core, boundary, and surface designs. It is evident from the difference in the scale of the two axes that the actual energy difference between the full and the sparse GMEC (ranges from 0.05 kcal/mol to 1.6 kcal/mol) is an order of magnitude smaller than the computed upper bound, which can be as high as 40 kcal/mol. As a result, Sparse A* returned the full GMEC relatively early, well before all conformations within the energy bound (calculated using Lemma 1 in S1 Text) of the sparse GMEC were enumerated.

The full GMEC was found within the first 20 conformations of the sparse GMEC for all 21 boundary design problems with energy cutoffs, and for 19 design problems with distance cutoffs. For the two remaining problems, ClpS protease adaptor protein (PDB id: 3DNJ) and bacterial ferredoxin protein (PDB id: 1IQZ), the full GMEC was the 168^th^ and 5062^nd^ conformation returned by Sparse A* respectively with distance cutoff *δ* = 7 Å. The rank of the full GMEC in the gap-free list of conformations enumerated by Sparse A* for all 21 boundary design problems are given in Table 3. For the 12 surface design problems, the full GMEC is within the first 30 conformations of the sparse GMEC for energy cutoffs, but for distance cutoffs, this number is on the order of a few hundred for some of the design problems. The rank of the full GMEC in the gap-free list of conformations enumerated by Sparse A* for all 12 surface design problems are given in Table 4.

**Table 3:** The full GMEC is usually within 30 conformations of the sparse GMEC for boundary designs. Rank of the full GMEC in the gap-free list of conformations generated by Sparse A* for 21 boundary protein design problems, with distance cutoffs *δ* = 7 Å and *δ* = 8 Å, and energy cutoffs *α* = 0.1 kcal/mol and *α* = 0.2 kcal/mol. Rank 1 indicates that the full and the sparse GMEC were identical.

PDB id	*δ* = 7 Å	*δ* = 8 Å	*α* = 0.1 kcal/mol	*α* = 0.2 kcal/mol
1G6X	1	1	1	1
1I27	1	1	1	1
1IQZ	5062	1448	1	17
1OAI	1	1	1	1
1OK0	2	2	1	2
1PSR	8	8	1	1
1TUK	1	1	1	1
1UCS	8	7	1	1
1VBW	7	5	1	4
2B97	6	1	1	3
2BWF	1	1	1	1
2CS7	11	11	1	8
2DSX	4	1	1	1
2HIN	1	1	1	1
2IC6	1	1	1	1
2ZXY	3	3	1	1
3DNJ	168	15	1	2
3FIL	2	2	1	1
3HFO	1	1	1	2
3G21	6	1	3	1
1C75	1	1	4	3

**Table 4:** The full GMEC is usually within 1000 conformations of the sparse GMEC for surface designs. Rank of the full GMEC in the gap-free list of conformations generated by Sparse A* for 12 surface protein design problems, with distance cutoffs *δ* = 7 Å and *δ* = 8 Å, and energy cutoffs *α* = 0.1 kcal/mol and *α* = 0.2 kcal/mol. Rank 1 indicates that the full and the sparse GMEC were identical.

PDB id	*δ* = 7 Å	*δ* = 8 Å	*α* = 0.1 kcal/mol	*α* = 0.2 kcal/mol
1C75	50	97	1	1
1G6X	1	1	1	1
1UCS	3	1	1	16
2CS7	37	64	1	4
2FMA	18	2	1	1
2HLR	6	6	1	1
2J8B	286	286	1	1
2O9S	840	943	1	2
2RH2	46	6	1	2
3FIL	57	1	1	1
1IQZ	2231	213	2	26
1V6P	14	12	5	22

Table 5 shows the rank of the full GMEC in the gap-free, in-order list enumerated after applying a distance cutoff *δ* = 7 Å for the six retrospective design problems discussed in the Section entitled “Retrospective validation against experimental data” (Table 1 and Fig 8), along with the search space of each design problem. Note that even after constraining the mutable residues to only two allowed amino acids, the search space size for these designs can be large. The search space of the largest retrospective design problem, a 19-residue design of the engrailed homeodomain dimer (PDB id: 2mg4), was 6.13 × 10^25^ conformations. Even in this case the rank of the full GMEC was merely 19 in the gap-free, in-order list, and our provable enumeration algorithm efficiently computes both. (For these experiments, computing 20 additional conformations after computing the sparse GMEC took less than 7.5 minutes.) The search space of the design problem that required the most enumeration, an 18-residue design of acyl phosphatase, was 6.64 × 10^24^, and enumerating 240 conformations to recover the full GMEC took less than 48 minutes. Across the six design problems, the median time to compute the sparse GMEC was 8.81 minutes, and the median time to compute the top 1000 conformations was 46.2 minutes. These times show how computing the top 1000 conformations is far less costly than computing 1000 GMECs. Therefore, using a provable algorithm to enumerate the 1000 lowest-energy conformations is in most cases very practical.

**Table 5:** The rank of the full GMEC is small for retrospective design problems, and the 1000 lowest-energy conformations can be enumerated quickly.

PDB id	Number of Residues	Number of Mutable Residues	Full GMEC Rank^a^	Search Space Size^b^ (conformations)	Sparse GMEC Time^c^ (minutes)	Full GMEC Time^d^ (minutes)	Time to 1000 Conformations^e^ (minutes)
1hz5	61	15	10	2.07 × 10^19^	21.0	25.1	60.7
1urn	64	16	2	1.87 × 10^20^	50.6	52.4	191
1enh	66	12	2	6.65 × 10^15^	0.230	0.254	20.3
2mg4	66	19	19	6.13 × 10^25^	1.01	1.52	19.2
2acy	98	18	240	6.64 × 10^24^	16.6	48.0	119
1vjq	73	26	3	4.10 × 10^18^	0.251	0.346	31.8

^a^Rank of the full GMEC in the gap-free list enumerated by A* when applying the distance cutoff *δ* = 7 Å

^b^Total number of possible conformations

^c^Time to compute the sparse GMEC

^d^Time to compute a gap-free list containing the full GMEC

^e^Time to compute a gap-free list of the 1000 lowest-energy conformations

Overall, the energy difference between sparse and full GMEC was small, and therefore the rank of the full GMEC in the gap-free list enumerated by Sparse A* was low. For all but one design problem (including the core, boundary, surface, and retrospective designs), the full GMEC was found by enumerating only the first 1000 conformations returned by Sparse A* (with both distance and energy cutoffs). This shows that even when limited time and memory prevent Sparse A* from provably enumerating the full GMEC (because of loose energy bounds), in practice the number of conformations that must be enumerated before Sparse A* returns the full GMEC can be small. This provides useful information that can be used to compute the full GMEC using Sparse A* for design problems where A* fails. This is highlighted by the three boundary and one surface design problem for which Sparse A* with distance cutoff *δ* = 7 Å (the cutoff which deleted the most of edges) returned the sparse GMEC, whereas A* ran out of 30 GB of memory before returning the full GMEC (orange points in Fig 2). Sparse A* returned the sparse GMEC along with a gap-free list of conformations for three boundary designs (heterogeneous nuclear ribonucleoprotein K (PDB id: 1ZZK), Beta-elicitin cinnamomin (PDB id: 2AIB), Dihydrofolate reductase type 2 (PDB id: 2RH2), and for one surface design of scorpion toxin protein (PDB id: 1AHO)). The number of conformations enumerated by Sparse A* was 47 for 1ZZK, 3029 for 2AIB, 46 for 2RH2, and 10,000 for 1AHO. Given the results that with distance cutoff *δ* = 7 Å the full GMEC can be found almost always within the first 30 conformations for boundary designs, and within 1000 conformations for surface designs, the gap-free list computed by Sparse A* for the above four protein design problems almost certainly contains the full GMEC. Because the number of conformations that must be enumerated by Sparse A* to find the full GMEC is usually small, the GMECs of both the full and the sparse residue interaction graphs can be computed by enumerating a gap-free, in-order list of conformations.

Note that this study relies critically on provable algorithms that are guaranteed to enumerate the GMEC followed by a gap-free list of conformations in order of increasing energy. Without these algorithms it would be difficult and perhaps even unsound to compare the results of computational protein design with and without sparse residue interaction graphs, since differences induced by the sparse model can not be deconvolved from differences stemming from undersampling or inadequate stochastic optimization. Moreover, the provable guarantees of Lemma 2 (S1 Text) would not be possible if the enumeration algorithm missed any low-energy conformations within the calculated energy window of the sparse GMEC.

It has been previously argued that crucial improvements to the energy function and input model (e.g. side-chain flexibility, backbone flexibility, and entropy) should, for reasons of computational complexity, be accompanied by novel algorithmic enhancements [39]. Hence, it is also important to distinguish design algorithms that only apply distance cutoffs to the energy function [24, 45–52] vs. algorithms that exploit the optimal substructure induced by sparse residue interaction graphs (via techniques such as dynamic programming) [53–60]. While algorithms that only modify the energy function and algorithms that effectively exploit the optimal substructure both benefit from the reduced effective search space of sparse residue interaction graphs, significant large-scale gains in computational efficiency (including reduced asymptotic time complexity) are not achieved by the former, whereas they are *guaranteed* by the latter. By developing new, efficient methodologies and algorithms (such as Energy-bounding enumeration, used in the Section entitled “Discussion”), larger, harder problems built on more sophisticated biophysical input models become tractable without any increase in hardware capability. So even though physical hardware power may not improve quickly enough to relieve the computational costs of protein design, algorithmic improvements can reduce previously difficult or even intractable tasks in protein design to well-understood problems for which a wealth of efficient algorithms already exist. One example is a recent paper [70], which reduces the problem of protein design with continuous side-chain flexibility to the well-understood problem of protein design with discrete rotamers, enabling designers to perform designs with continuous side-chain flexibility as efficiently as they could previously perform discrete rigid rotamer designs.

### Conclusion

In this paper, we implemented a variant of the A* search algorithm in our lab’s open source protein design package osprey, for protein design with sparse residue interaction graphs. We ran A* and Sparse A* on 136 different protein design problems involving core, boundary, and surface residues and analyzed the effects of using various distance and energy cutoffs. We compared the energies and sequences of the full GMEC returned by A* vs. the sparse GMEC returned by Sparse A*, and found that distance cutoffs, especially in surface and boundary designs, can lead to significant sequence differences between the full and the sparse GMEC. Our analysis indicates that the effects of distance cutoffs range from introducing no sequence differences in core designs, to sequence differences in almost all surface designs. By comparison, the effects of energy cutoffs are similar across core, boundary, and surface designs. In addition, we show examples of protein design problems in which neglecting long-range interactions alters local interactions. Furthermore, we performed retrospective designs for proteins with experimentally measured data, and our analysis of sequence differences between the full and the sparse GMEC indicates that it is not readily apparent if the sparse or full GMEC predicts mutations that perform better *in vitro*.

The sequence differences between the full and the sparse GMEC occur even though the energy differences between these GMECs are small. While these errors are severe, we provided a way to overcome these discrepancies. We used a provable, ensemble-based algorithm and showed that the full GMEC was found within the first 1000 conformations returned for all but one of our design problems. Because the number of conformations that must be enumerated to find the full GMEC is usually small, we can take advantage of the reduced search space provided by sparse residue interaction graphs and still efficiently compute both the full and the sparse GMEC. To do this, we compute a gap-free, in-order list of conformations. The gap-free list of low-energy conformations returned by Sparse A* is guaranteed to contain the full GMEC, and we show it takes only polynomial additional time to find the full GMEC (Lemma 2 in S1 Text). For 3 boundary and 1 surface design problem where A* failed to return even a single conformation, Sparse A* not only computed the sparse GMEC, but also enumerated a gap-free list of conformations that almost certainly contains the full GMEC. This provides a novel way for provable, ensemble-based algorithms and sparse residue interaction graphs to compute not only the sparse GMEC, but also the full GMEC for previously intractable design problems.

Previous studies have found that computational structure-based protein design protocols are often susceptible to forcefield inaccuracies (particularly hydrogen bonding and electrostatics) [71]. Distance cutoffs are widely used because interaction energy decreases with distance, which is relatively inexpensive (compared with energy cutoffs) to calculate. The underlying assumption is that beyond a certain distance, the interaction energy is negligible. Our results show that this is, in fact, not always true: these seemingly negligible interaction energies can add up, leading to significant sequence differences and changes in local residue interactions that can alter not only the structure, but also the function of the predicted protein. Therefore by using distance cutoffs, protein designers have been neglecting significant pairwise interactions, which may compromise the accuracy of their predictions. Our paper is the first large scale study of the magnitude and consequences of distance cutoffs and their effects. On the positive side, we showed that by combining sparse residue interaction graphs with provable, ensemble-based algorithms, we provide a way to overcome this inaccuracy. Therefore, by using provable algorithms in the manner we described, protein designers can continue to reap the benefits of distance cutoffs without worrying about loss in accuracy. The gap-free list of conformations generated will include the sequence of both the sparse and full GMEC, allowing both sequences to be inspected and tested. We believe this is a notable improvement over traditional protocols, which require designers to commit beforehand to either the sparse or full interaction model. Our work simultaneously exposes potentially significant experimental inaccuracies in the input model and provides novel methodology to address these inaccuracies directly.

## Supporting information

S1 Text Supplementary theory: Proofs for Lemma 1 and Lemma 2. (PDF)

S2 Text Supplementary theory and methods: Sparse residue interaction graphs. (PDF)

S3 Text Full details of computational experiments performed. (PDF)

S1 Fig Distance cutoffs prune a larger percentage of edges than energy cutoffs in boundary and surface design problems. Number of unpruned conformations left after DEE vs. the percentage of edges deleted from the residue interaction graph. Two data points are plotted for each design problem: with distance cutoff *δ* = 7 Å (blue), and energy cutoff *α* = 0.2 kcal/mol (red). (a) 62 core design problems, (b) 46 boundary design problems, and (c) 28 surface design problems.
(TIF)

S2 Fig Distance cutoffs introduce amino acid changes in more residues than energy cutoffs. Amino acid identities of residues in the full GMEC which were mutated in the sparse GMEC, for boundary and surface protein design problems with distance cutoff *δ* = 7 Å and energy cutoff *α* = 0.2 kcal/mol. The number in parenthesis indicates the cumulative number of residues across all design problems for which that amino acid was different in the sparse GMEC.
(TIF)

S1 Table Sequence differences between GMECs for boundary designs. Sequence differences between the full and the sparse GMEC for boundary design problems, for distance cutoff *δ* = 7 Å and energy cutoff *α* = 0.2 kcal/mol. A dash (“−”) indicates that the amino acid identity was the same in the full and the sparse GMEC.
(PDF)

S2 Table Sequence differences between GMECs for surface designs. Sequence differences between the full and the sparse GMEC for surface design problems, for distance cutoff *δ* = 7 Å and energy cutoff *α* = 0.2 kcal/mol. A dash (“−”) indicates that the amino acid identity was the same in the full and the sparse GMEC.
(PDF)

S3 Table Sequence correlation between designed mutant and wild type. The table shows the sequences of the sparse and full GMEC. Residues at which the sparse or full GMEC has the same amino acid identity as the thermostabilized mutant are in bold. Residues at which the sparse or full GMEC has the same amino acid identity as the less stable wild-type are not in bold.
(PDF)
