# Supplementary material for: A critical analysis of computational protein design with sparse residue interaction graphs
Source: PLoS Comput Biol. 2017 Mar 30;13(3):e1005346. doi: 10.1371/journal.pcbi.1005346 (PMC5391103; doi:10.1371/journal.pcbi.1005346)
Supplement: S1 Text — (PDF) [file pcbi.1005346.s001.pdf]

S1 Text

Supplementary Theory: Computing the Sparse and Full GMEC with Sparse A*

In Sec. 4.1, we provided high level intuition showing how Sparse A* can be used to compute both the sparse and full GMEC. These statements are supported by mathematical guarantees, which show that the full GMEC is contained in the gap-free list enumerated by Sparse A*, and that it is efficient to compute it from that list. In this document we provide two Lemmas and their proofs. Lemma 1 proves an upper bound on the absolute difference in sparse energy between the sparse and full GMEC, and Lemma 2 gives the time complexity to compute the full GMEC from a gap-free list guaranteed to contain the full GMEC. We then describe how these two proofs are sufficient to compute both the sparse and full GMEC with Sparse A*. Finally, we briefly describe a recent provable algorithm [1], which uses concepts from dynamic programming to exploit the optimal substructure induced by sparse graphs, and achieve asymptotic time complexity significantly better than the worst-case time complexity of any algorithm using the full graph.

Given a distance cutoff δ and an energy cutoff α, a sparse residue interaction graph G' can be generated by deleting the set of edges $\mathcal{E}'$ from the full residue interaction graph G. To review, we repeat the definitions of *full energy* and *sparse energy* below.

The full energy $E(c)$ of a conformation c is defined to be

$$E(c) = \sum_{j \geq i} (c_i, c_j). \tag{1}$$

The sparse energy $E'(c)$ of c is defined to be

$$E'(c) = \sum_{j \geq i} E(c_i, c_j) - \sum_{\substack{j > i \\ (i,j) \in \mathcal{E}'}} E(c_i, c_j). \tag{2}$$

Because certain interaction energies are omitted from the sparse residue interaction graph, the full GMEC c^* (conformation with minimum full energy in Eq. 1) and the sparse GMEC c' (conformation with minimum sparse energy in Eq. 2) may be different. It would be useful to upper bound the energy difference between the full and the sparse GMEC. If r_i and s_j denote rotamer r and s at residues i and j respectively, let E'_{max} and E'_{min} be defined as the respective sums of the maximal and minimal energies contributed by each edge $e \in \mathcal{E}'$ eliminated from G.

$$E'_{max} = \sum_{\substack{j>i \\ (i,j)\in\mathcal{E}'}} \max_{r,s} E(r_i, s_j). \tag{3}$$

$$E'_{min} = \sum_{\substack{j>i \\ (i,j)\in\mathcal{E}'}} \min_{r,s} E(r_i, s_j). \tag{4}$$

The following lemma bounds the difference in the full and sparse energies of the full and the sparse GMEC. For completeness, we provide this proof, which is adapted from [1].

Lemma 1. *Given a full residue interaction graph G, a sparse residue interaction graph G', the full GMEC c^* and sparse GMEC c', the difference in sparse energy of c^* and c' is bounded by the relationship $|E'(c^*) - E'(c')| \leq E'_{max} - E'_{min}$.*

Proof. By definition, the full GMEC c^* has the minimum full energy and the sparse GMEC c' has the minimum sparse energy. Hence,

$$E(c^*) \leq E(c'). \tag{5}$$

$$E'(c') \leq E'(c^*). \tag{6}$$

Define $\delta'(c^*) = \sum_{\substack{j>i \\ (i,j)\in\mathcal{E}'}} E(c_i^*, c_j^*)$ to be the difference between the full energy and sparse energy of the full GMEC c^*. $\delta'(c')$ is defined accordingly. By definition both $\delta'(c^*)$ and $\delta'(c')$ lie in between E'_{max} and E'_{min}. The sparse energy can now be expressed in terms of the full energy as follows:

$$E(c^*) = E'(c^*) + \delta'(c^*). \tag{7}$$

$$E(c') = E'(c') + \delta'(c'). \tag{8}$$

Substituting Eq. (7) and Eq. (8) in Eq. (5), we obtain

$$E'(c^*) + \delta'(c^*) \leq E'(c') + \delta'(c'). \tag{9}$$

Substituting $\delta'(c^*)$ by E'_{min}, and $\delta'(c')$ by E'_{max} maintains the inequality:

$$E'(c^*) + E'_{min} \leq E'(c') + E'_{max}. \tag{10}$$

Rearranging,

$$E'(c^*) - E'(c') \leq E'_{max} - E'_{min}. \tag{11}$$

As from Eq. (6), $E'(c^*) - E'(c') \geq 0$, which means

$$|E'(c^*) - E'(c')| \leq E'_{max} - E'_{min}. \tag{12}$$

□

Lemma 1 holds for any distance or energy cutoff used to generate the sparse residue interaction graph, although eliminating more edges will increase the energy bounds. Although the bounds given in Lemma 1 are often loose, it is important to know that the energy difference between the full and the sparse GMEC can always be bounded. This allows us to use provable, ensemble-based algorithms that enumerate a gap-free list of conformations to compute both the sparse and full GMEC in a single gap-free list. This gap-free list is guaranteed to contain both GMECs.

The Sparse A* algorithm retains all the guarantees of the A* search algorithm. This means that the first conformation enumerated by Sparse A* is guaranteed to be the sparse GMEC, and that Sparse A* can enumerate a gap-free list of conformations, within an energy window E_w of the sparse GMEC, in order of sparse energy. This property of Sparse A* can be used to enumerate not only the sparse GMEC, but also the full GMEC. When $E_w \geq E'_{max} - E'_{min}$, the upper bound on the difference in sparse energy between the full and the sparse GMEC as calculated by Lemma 1, the gap-free list enumerated by Sparse A* is guaranteed to contain the full GMEC. This is true in general for any ensemble-based provable algorithm [1, 2]. This is proved in the following lemma.

Lemma 2. *Given a protein design problem with n residues, the gap-free list of conformations generated by Sparse A* is guaranteed to contain the full GMEC if $E_w \geq E'_{max} - E'_{min}$, and can be found in additional $O(kn^2)$ time, where k is the number of conformations generated.*

Proof. After the Sparse A* run ends and it returns k conformations within the specified energy window E_w, the full energy for each of these k conformations is recalculated according to Eq. (1). Since the energy of a conformation can be computed in $O(n^2)$ time, recomputing the energy for k conformations can be done in $O(kn^2)$ time. Next, the conformation with the minimum full energy (full GMEC) within the list of k conformations can be found in $O(k)$ time. □

The fact that Sparse A* can enumerate both the sparse and the full GMEC means that we no longer have to worry about which of the two sequences (the full or the sparse GMEC) will predict the desired functional mutations. One can generate the sparse residue interaction graph, calculate the upper bound on the energy difference,

and run Sparse A* to return a gap-free list of conformations that is guaranteed to return the full GMEC. The list of conformations returned by Sparse A* can then be re-ranked based on the full energy to get the full GMEC. Once both the full and the sparse GMEC are found, they can be evaluated based on more sophisticated methods for energy calculation [3, 4], or any other method that seems pertinent to the designer. Note that both the full and the sparse GMEC are guaranteed to be found only when using provable ensemble-based algorithms that are guaranteed not to miss any conformation within the specified energy window. The re-ranking can be done relatively quickly (Lemma 1), when the number of conformations that need to be generated by Sparse A* to find the full GMEC is not large. Both the full and the sparse GMEC were found for most of the design problems used in this study, and this is discussed in Section 4.2.

It has been previously argued that crucial improvements to the energy function and input model (e.g. side-chain flexibility, backbone flexibility, and entropy) should, for reasons of computational complexity, be accompanied by novel algorithmic enhancements [5]. Hence, it is also important to distinguish design algorithms that only apply distance cutoffs to the energy function [6, 7, 8, 9, 10, 11, 12, 13, 14] vs. algorithms that exploit the optimal substructure induced by sparse residue interaction graphs (via techniques such as dynamic programming) [15, 16, 17, 18, 19, 1, 20]. While algorithms that only modify the energy function and algorithms that effectively exploit the optimal substructure both benefit from the reduced effective search space of sparse residue interaction graphs, significant large-scale gains in computational efficiency (including reduced asymptotic time complexity) are not achieved by the former, whereas they are *guaranteed* by the latter. For example, a recent study reduces the worst-case time complexity of computing the sparse GMEC from being exponential in n (i.e., $O(q^n)$) to being merely exponential in the branch-width w of the sparse residue interaction graph (i.e., $O(q^{\frac{3}{2}w})$) [1]. In practice, that study found that w was frequently much smaller than n. Therefore, precomputing the sparse residue interaction graph (in $O(q^2n^2)$ time) to evaluate all q^2 possible pairwise energies for all $\binom{n}{2}$ possible residue pairs can significantly reduce the worst-case time complexity to compute the sparse GMEC.

References

1. Jou JD, Jain S, Georgiev IS, Donald BR. BWM*: A Novel, Provable, Ensemble-based Dynamic Programming Algorithm for Sparse Approximations of Computational Protein Design. J Comput Biol. 2016;23(6):413–424.

2. Roberts KE, Gainza P, Hallen MA, Donald BR. Fast gap-free enumeration of conformations and sequences for protein design. Proteins: Structure, Function, and Bioinformatics. 2015 Oct;83(10):1859–1877.

3. Hallen MA, Gainza P, Donald BR. Compact Representation of Continuous Energy Surfaces for More Efficient Protein Design. Journal of Chemical Theory and Computation. 2015;11(5):2292–2306.

4. Hallen MA, Jou JD, Donald BR. LUTE (Local Unpruned Tuple Expansion): Accurate Continuously Flexible Protein Design with General Energy Functions and Rigid Rotamer-Like Efficiency. J Comput Biol. 2016; Epub ahead of print.

5. Gainza P, Nisonoff HM, Donald BR. Algorithms for protein design. Current Opinion in Structural Biology. 2016;39:16–26.

6. Jones DT. De novo protein design using pairwise potentials and a genetic algorithm. Protein Science. 1994 Apr;3(4):567–574.

7. Koehl P, Delarue M. Application of a Self-consistent Mean Field Theory to Predict Protein Side-chains Conformation and Estimate Their Conformational Entropy. Journal of molecular biology. 1994 Jun;239(2):249–275.

8. Desjarlais JR, Handel TM. De novo design of the hydrophobic cores of proteins. Protein Science. 1995 Oct;4(10):2006–2018.

9. Jiang X, Farid H, Pistor E, Farid RS. A new approach to the design of uniquely folded thermally stable proteins. Protein Science. 2000 Feb;9(02):403–416.

10. Desmet J, Spriet J, Lasters I. Fast and accurate side-chain topology and energy refinement (FASTER) as a new method for protein structure optimization. Proteins: Structure, Function, and Bioinformatics. 2002 Jul;48(1):31–43.

11. Kortemme T, Morozov AV, Baker D. An Orientation-dependent Hydrogen Bonding Potential Improves Prediction of Specificity and Structure for Proteins and Protein–Protein Complexes. Journal of molecular biology. 2003 Feb;326(4):1239–1259.

12. Kingsford CL, Chazelle B, Singh M. Solving and analyzing side-chain positioning problems using linear and integer programming. Bioinformatics. 2005 Apr;21(7):1028–1039.

13. Leaver-Fay A, Tyka M, Lewis SM, Lange OF, Thompson J, Jacak R, et al. ROSETTA3: an object-oriented software suite for the simulation and design of macromolecules. In: Methods in Enzymology. Methods in Enzymology; 2011. p. 540–574.

14. Privett HK, Kiss G, Lee TM, Blomberg R, Chica RA, Thomas LM, et al. Iterative approach to computational enzyme design. Proceedings of the National Academy of Sciences. 2012 Mar;109(10):3790–3795.

15. Canutescu AA, Shelenkov AA, Dunbrack RL. A graph-theory algorithm for rapid protein side-chain prediction. Protein Science. 2003 Sep;12(9):2001–2014.

16. Leaver-Fay A, Kuhlman B, Snoeyink J. An adaptive dynamic programming algorithm for the side chain placement problem. Pacific Symposium on Biocomputing. 2005;10:16–27.

17. Xu J, Berger B. Fast and accurate algorithms for protein side-chain packing. Journal of the ACM (JACM). 2006 Jul;53(4):533–557.

18. Krivov GG, Shapovalov MV, Dunbrack RL. Improved prediction of protein side-chain conformations with SCWRL4. Proteins: Structure, Function, and Bioinformatics. 2009 Dec;77(4):778–795.

19. Xu J. Rapid Protein Side-Chain Packing via Tree Decomposition. In: Research in Computational Molecular Biology, Lecture Notes in Computer Science. vol. 3500. Proceedings of the Annual International Conference on Research in Computational Molecular Biology (RECOMB), Cambridge, May 14-18, 2005. Springer-Verlag (Berlin); 2005. p. 423–439.

20. Zhou Y, Wu Y, Zeng J. Computational Protein Design Using AND/OR Branch-and-Bound Search. J Comput Biol. 2016;23(6):439–451.
