# Supplementary material for: A critical analysis of computational protein design with sparse residue interaction graphs
Source: PLoS Comput Biol. 2017 Mar 30;13(3):e1005346. doi: 10.1371/journal.pcbi.1005346 (PMC5391103; doi:10.1371/journal.pcbi.1005346)
Supplement: S2 Text — (PDF) [file pcbi.1005346.s002.pdf]

S2 Text

Supplementary Theory and Methods: Sparse Residue Interaction Graphs

In the section entitled "Definitions related to Sparse Residue Interaction Graphs", we provide a high-level intuition for the mathematical basis of sparse residue interaction graphs, and their corresponding energy functions. For the proofs of Lemma 1 and Lemma 2 in **S6 Text**, however, precise definitions are useful. In this document, we provide formal definition of a residue interaction graph, the sparse residue interaction graph, and the corresponding GMECs computed using such interaction graphs. We also provide mathematical details for the cutoff criteria used to prune pairwise interactions from a residue interaction graph.

Each protein design problem is defined by its input model, namely, the input protein structure, the mutable residues, the allowed amino acids at each mutable residue, and allowed side-chain conformations[1]. Such a protein design problem can be represented as an undirected complete graph $G = (V, \mathcal{E})$, where V represents all mutable residue positions ($|V| = n$), and $\mathcal{E}$ represents all possible pairwise residue interactions ($|\mathcal{E}| = \binom{n}{2}$).

For a protein design with n mutable residues, let i and j denote two residues, and for a given conformation c, let $E(c_i, c_j)$ be the pairwise energy between them. When the self-energy of the residues can be included in the pairwise energy terms, the energy of a given conformation c is represented in Eq. (1):

$$E(c) = \sum_{j \geq i} (c_i, c_j). \tag{1}$$

We will refer to the graph G as a *full residue interaction graph*, the energy of any conformation calculated using Eq. (1) as its *full energy*, and the protein conformation that minimizes the full energy over all possible rotamer assignments as the *full GMEC*.

Let $\mathcal{E}$' be the set of edges deleted from G to generate a *sparse residue interaction graph*. If r_i and s_j represent rotamers r and s at residues i and j respectively, an edge between i and j is deleted from the graph G if it meets any the following two conditions:

[1] Protein design formulations that consider additional side-chain flexibility [1, 2], backbone flexibility [3–5], free energy calculations [6, 7], or more accurate energy functions [8] have been developed. Nevertheless most of them call as a subroutine the simplified model discussed in this paper, which can be viewed as a core calculation common to most protein design software. Hence, the accuracy of this computation bounds the accuracy of the overall design.

- $d_{\min}(i,j) > \delta$, where $d_{\min}(i,j) = \min_{r,s} d(r_i, s_j)$ is the closest Euclidean distance between any two atoms in residues i and j when all rotamer combinations for these two residues are considered; δ is the distance cutoff parameter;
- $E_{\max}(i,j) < \alpha$, where $E_{\max}(i,j) = \max_{r,s} |E(r_i, s_j)|$ is the maximum energy (in absolute value) between residues i and j when all rotamer combinations for these two residues are considered; α is the interaction energy cutoff parameter.

With appropriate distance and energy cutoffs, a set of edges $\mathcal{E}'$ are deleted from the residue interaction graph G, producing the sparse residue interaction graph $G' = (V, \mathcal{E} - \mathcal{E}')$. The energy of a conformation c with respect to G' can now be written as:

$$E'(c) = \sum_{j \geq i} E(c_i, c_j) - \sum_{\substack{j>i \\ (i,j)\in\mathcal{E}'}} E(c_i, c_j). \tag{2}$$

We will refer to the energy of any conformation as calculated using Eq. 2 as its *sparse energy*, and the protein conformation that minimizes the sparse energy over all possible rotamer assignments as the *sparse GMEC*. Note that because certain interaction energies are omitted from the sparse residue interaction graph, the full GMEC (conformation with minimum full energy in Eq. 1) and the sparse GMEC (conformation with minimum sparse energy in Eq. 2) may be different.

References

1. Gainza P, Roberts KE, Donald BR. Protein Design Using Continuous Rotamers. PLoS computational biology. 2012 Jan;8(1):e1002335.

2. Georgiev I, Lilien RH, Donald BR. The minimized dead-end elimination criterion and its application to protein redesign in a hybrid scoring and search algorithm for computing partition functions over molecular ensembles. Journal of Computational Chemistry. 2008 Jul;29(10):1527–1542.

3. Georgiev I, Donald BR. Dead-end elimination with backbone flexibility. Bioinformatics. 2007 Jul;23(13):i185–94.

4. Georgiev I, Keedy D, Richardson JS, Richardson DC, Donald BR. Algorithm for backrub motions in protein design. Bioinformatics. 2008 Jul;24(13):i196–204.

5. Hallen MA, Keedy DA, Donald BR. Dead-end elimination with perturbations (DEEPer): A provable protein design algorithm with continuous sidechain and backbone flexibility. Proteins: Structure, Function, and Bioinformatics. 2013 Jan;81(1):18–39.

6. Lilien RH, Stevens BW, Anderson AC, Donald BR. A novel ensemble-based scoring and search algorithm for protein redesign and its application to modify the substrate specificity of the gramicidin synthetase a phenylalanine adenylation enzyme. Journal of Computational Biology. 2005 Jul;12(6):740–761.

7. Georgiev I, Lilien RH, Donald BR. The minimized dead-end elimination criterion and its application to protein redesign in a hybrid scoring and search algorithm for computing partition functions over molecular ensembles. Journal of Computational Chemistry. 2008 Jul;29(10):1527–1542.

8. Hallen MA, Gainza P, Donald BR. Compact Representation of Continuous Energy Surfaces for More Efficient Protein Design. Journal of Chemical Theory and Computation. 2015;11(5):2292–2306.
