# Supplementary material for: A critical analysis of computational protein design with sparse residue interaction graphs
Source: PLoS Comput Biol. 2017 Mar 30;13(3):e1005346. doi: 10.1371/journal.pcbi.1005346 (PMC5391103; doi:10.1371/journal.pcbi.1005346)
Supplement: S3 Text — (PDF) [file pcbi.1005346.s003.pdf]

S3 Text

In this document we provide complete details of the computational experiments conducted.

In all experiments, the DEE pruning stage was followed by either A* with $E_w = 0$ to get the full GMEC, or the following two steps to generate the sparse GMEC and gap-free list of conformations: 1) Sparse residue interaction graph generation and calculation of energy bounds E_b (Lemma 1) using a user-defined distance cutoff δ or energy cutoff α, and 2) Sparse A* run with $E_w = E_b$ to generate the sparse GMEC and a gap-free list of conformations. Sparse A* was run until all conformations within E_w of the sparse GMEC were returned, or until the number of conformations returned was 10,000. For each design problem, Sparse A* was run four times using the following distance or energy cutoffs:

- $\delta = 8$ Å;
- $\delta = 7$ Å;
- $\alpha = 0.1$ kcal/mol;
- $\alpha = 0.2$ kcal/mol.

To further investigate how the predicted sequence and conformation differences between the full and the sparse GMEC would correlate with experimental results, we performed retrospective validation against full-sequence designs from the literature, for which the designed mutants were experimentally determined to have improved

thermostability over the wild type [1–4]. The designed dimer of the engrailed homeodomain [3] (PDB id: 2mg4) was an example of the design of a novel multimeric fold, and is additionally valuable as an example of how sparse residue interaction graphs affect more ambitious changes in the structure of the protein. Each example taken from the literature consisted of a design with an input structure and experimentally measured melting point data showing a more thermostable designed mutant compared to the wild type sequence. For our computational experiments, hydrogen atoms were added to the input structure with Reduce [5] and incomplete residues were mutated to alanine. The input structures were then minimized with Sander [6] before use. Consecutive residues of one or more adjacent secondary structures were designed to either retain the their wild-type identify or mutate to amino acid identity of the thermostabilized mutant. To account for large backbone changes for the example of the designed dimer of the engrailed homeodomain [3] (PDB id: 2mg4) the backbone of the mutant was used, and the sequence of the mutant was retained at all immutable residues. For the design of human procarboxypeptidase A2 [4], a side-chain placement problem was performed instead, and all residues retained the amino acid identity of the mutant (PDB id: 1vjq). As described above, DEE pruning was followed by A* search to compute the full GMEC or Sparse A* to compute the sparse GMEC. The number of residues designed varied from 10-19 residues and a distance cutoff of $\delta =$ 7 Å was applied to generate the sparse residue interaction graph. The full and the sparse GMEC were then correlated against the measured melting point data.

All computations were performed on Intel Xeon processor nodes with number of cores ranging from 8-48, and processor speeds ranging from 2.40-2.66GHz. Each core

design was given 10GB of memory, and each boundary and surface design was given 30 GB. The energy function consisted of the AMBER van der Waals and electrostatic terms [7] and the EEF1 pairwise implicit solvation model [8]. A distance-dependent dielectric of 6 and a solvation energy scaling factor of 0.05 were used. The atomic van der Waals radii were scaled by a factor of 0.95. Additionally, amino acid-specific reference energies were computed using the lowest computed intra-rotamer energy for each amino acid type among all mutable residue positions (similarly to [9]). All designs were done keeping the backbone fixed and modeling side-chain flexibility using the modal values of rotamers from the Penultimate rotamer library [10].

Table A. PDB ids, chain, and number of mutable residues for 62 core design problems for which A* and Sparse A* were run. The table also includes 5 additional design cases where DEE was run but returned only one conformation (indicated by †), and hence A* and Sparse A* runs were not performed.

Protein Structure	PDB id	Chain	Number of Residues	Number of Mutable Residues
Scorpion Toxin protein	1aho	A	64	13
Cytochrome C-553 from B. Pasteurii	1c75	A	71	12
ATX1 Metallochaperone	1cc8	A	73	14
Bucadin Roxin from Malayan Krait *dagger*	1f94	A	63	6
Maize Lipid-Transfer Protein	1fk5	A	93	7

Continued on next page

Table A – *Continued from previous page*

Protein Structure	PDB id	Chain	Number of Residues	Number of Mutable Residues
Bovine Pancreatic Trpisin Inhibitor	1g6x	A	58	11
C-Terminal domain of the RAP74 subunit of human TFIIF	1i27	A	73	14
Oxidized [4Fe-4S] Ferredoxin from B. Thermoprotelyticus	1iqz	A	81	15
TRP Repressor	1jhg	A	101	14
NapB subunit of the periplasmic nitrate reductase from H. influenzae.	1jni	A	123	5
Granulysin from human cytolytic T lymphocytes	1l9l	A	74	15
Ribonuclease from Streptomyces Aureofacinens	1lni	A	192	15
Tetraheme cytochrome c from Shewanella oneidensis MR1 †	1m1q	A	91	4
Cytochrome b5 domain of human sulfite oxidase	1mj4	A	82	12

Continued on next page

Table A – *Continued from previous page*

Protein Structure	PDB id	Chain	Number of Residues	Number of Mutable Residues
HI0828, a Hypothetical Protein from Haemophilus influenzae	1mwq	A	202	14
TAP UBA Domain and FXFG Nucleoprotein peptide	1oai	A	68	14
Tendamistat	1ok0	A	74	15
Human Psoriasin (S100A7)	1psr	A	200	14
syntenin PDZ2	1r6j	A	82	14
Apo acyl carrier protein from E. coli	1t8k	A	77	15
Type 2 non specific lipid transfer protein from wheat	1tuk	A	67	14
C-term. part of TonB	1u07	A	180	15
N-terminally truncated human APEP-1	1u2h	A	99	15
Dissimilatory sulfite reductase D (DsrD)	1ucr	A	156	11

Continued on next page

Table A – *Continued from previous page*

Protein Structure	PDB id	Chain	Number of Residues	Number of Mutable Residues
Type III Antifreeze Protein RD1 from an Antarctic Eel Pout	1ucs	A	64	14
Transcriptional Coactivator DCOH	1usm	A	80	13
Cobrotoxin †	1v6p	A	62	8
Bitter Gourd Trypsin Inhibitor	1vbw	A	68	12
FYVE Domain of VPS27P protein from Saccharomyces Cerevisiae	1vfy	A	73	10
Streptomyces castaneoglobisporus tyrosinase complexed with a caddie protein	1wxc	B	415	12
ygfY from Escherichia coli	1x6i	A	182	14
Zb domain from the RNA editing enzyme ADAR1	1xmk	A	79	15
The third KH domain of hnRNP K	1zzk	A	82	12

Continued on next page

Table A – *Continued from previous page*

Protein Structure	PDB id	Chain	Number of Residues	Number of Mutable Residues
Beta-cinnamomin in complex with ergosterol	2aib	A	196	10
Hydrophobin HFBII	2b97	A	142	12
Lectin from Ralstonia Solanacearum	2bt9	A	270	13
UBL Domain of DSK2 from S. Cerevisiae	2bwf	A	154	12
Dodecin with Flavin	2cc6	A	68	12
Second and third Fibronectin Type I Module Pair	2cg7	A	90	12
CBM31 from beta-1,3-xylanase	2cov	D	624	13
S. pneumoniae PhtA histidine triad domain	2cs7	A	165	14
Chorismate Mutase (Form I) from Thermus Thermophilus HB8	2d8d	A	180	5
LDL Receptor Ligand-Binding Modules 3-4 and the Receptor Associated Protein (RAP) †	2fcw	B	109	10

Continued on next page

Table A – *Continued from previous page*

Protein Structure	PDB id	Chain	Number of Residues	Number of Mutable Residues
Rubredoxin from Desulfovibrio gigas	2dsx	A	52	9
Colicin E5	2fhz	A	217	15
Alzheimer's Amyloid Precursor Protein (APP) Copper Binding Domain	2fma	A	59	9
Efb-C from Staphylococcus aureus	2gom	A	122	13
N-terminal Domain of Ribosomal Protein L9 (NTL9) K12M	2hba	A	104	10
N15: an ortholog of lambda Cro	2hin	A	142	11
Extracellular Domain of the Type II BMP Receptor	2hlr	A	100	10
HIV-1 protease V32I mutant with TMC114 (darunavir) inhibitor	2hs1	A	198	14
The Coiled-coil Domain (residues 1-75) of the Sin Nombre Virus Nucleocapsid Protein	2ic6	A	156	13

Continued on next page

Table A – *Continued from previous page*

Protein Structure	PDB id	Chain	Number of Residues	Number of Mutable Residues
Human CD59	2j8b	A	79	9
The second SH3 domain from ponsin	2o9s	A	67	14
N-terminal domain of AhrC	2p5k	A	64	11
CusF-Ag(I) residues 10-88 from Escherichia coli	2qcp	X	80	15
Scytovirin †	2qsk	A	95	10
DHFR R-67	2rh2	A	62	14
Rat Alpha Crystallin Domain	2wj5	A	101	15
Cytochrome c555 from Aquifex aeolicus	2zxy	A	87	14
High-potential iron-sulfur protein from Thermochromatium tepidum	3a38	A	83	13

Continued on next page

Table A – *Continued from previous page*

Protein Structure	PDB id	Chain	Number of Residues	Number of Mutable Residues
Caulobacter crescentus ClpS protease adaptor protein in complex with a N-end rule peptide	3dnj	A	190	12
GB1	3fil	A	112	14
C-terminal domain of the Rous Sarcoma Virus capsid protein	3g21	A	77	15
An Hfq protein from Synechocystis sp.	3hfo	A	210	10
Cold shock protein E from Salmonella typhimurium	3i2z	A	142	14
Arm-type binding domain of HPI integrase	3jtz	A	88	14

Table B. PDB ids, chain, and number of mutable residues for 46 boundary design problems.

Protein Structure	PDB id	Chain	Number of Residues	Number of Mutable Residues
Scorpion Toxin protein	1aho	A	64	16
Cytochrome C-553 from B. Pasteurii	1c75	A	71	17
ATX1 Metallochaperone	1cc8	A	73	20
Bucadin, Toxin isolated from the Malayan Krait	1f94	A	63	16
Maize Lipid-Transfer Protein	1fk5	A	93	19
Bovine Pancreatic Trpisin Inhibitor	1g6x	A	58	12
C-Terminal domain of the RAP74 subunit of human TFIIF	1i27	A	73	14
Oxidized [4Fe-4S] Ferredoxin from B. Thermoprotelyticus	1iqz	A	81	19
Granulysin from human cytolytic T lymphocytes	1l9l	A	74	18
TAP UBA Domain and FXFG Nucleoprotein Peptide	1oai	A	68	10

Continued on next page

Table B – *Continued from previous page*

Protein Structure	PDB id	Chain	Number of Residues	Number of Mutable Residues
Tendamistat	1ok0	A	74	19
Human Psoriasin	1psr	A	200	17
Apo acyl carrier protein from E. coli	1t8k	A	77	17
Type 2 non specific lipid transfer protein from wheat	1tuk	A	67	11
Dissimilatory sulfite reductase D (DsrD)	1ucr	A	156	19
Type III Antifreeze Protein RD1 from an Antarctic Eel Pout	1ucs	A	64	17
Transcriptional Coactivator DCOH	1usm	A	80	17
Cobrotoxin	1v6p	A	124	15
Bitter Gourd Trypsin Inhibitor	1vbw	A	68	14
FYVE Domain of VPS27P Protein from S. Cerevisiae	1vfy	A	73	18

Continued on next page

Table B – *Continued from previous page*

Protein Structure	PDB id	Chain	Number of Residues	Number of Mutable Residues
Streptomyces castaneoglobisporus tyrosinase complexed with a caddie protein	1wxc	B	415	17
The third KH domain of hnRNP K	1zzk	A	82	19
Beta-cinnamomin in complex with ergosterol	2aib	A	196	18
Hydrophobin HFBII	2b97	A	142	12
UBL Domain of DSK2 from S. Cerevisiae	2bwf	A	154	19
S. pneumoniae PhtA histidine triad domain	2cs7	A	165	12
Chorismate Mutase (Form I) from Thermus Thermophilus HB8	2d8d	A	180	15
Rubredoxin from Desulfovibrio gigas	2dsx	A	52	12

Continued on next page

Table B – *Continued from previous page*

Protein Structure	PDB id	Chain	Number of Residues	Number of Mutable Residues
Alzheimer's Amyloid Precursor Protein (APP) Copper Binding Domain	2fma	A	59	17
Efb-C from Staphylococcus aureus	2gom	A	122	17
N-terminal Domain of Ribosomal Protein L9 (NTL9) K12M	2hba	A	104	13
N15: an ortholog of lambda Cro	2hin	A	142	11
Extracellular Domain of the Type II BMP Receptor	2hlr	A	100	19
The Coiled-coil Domain (residues 1-75) of the Sin Nombre Virus Nucleocapsid Protein	2ic6	A	156	12
Human CD59	2j8b	A	79	18
The second SH3 domain from ponsin	2o9s	A	67	17
N-terminal domain of AhrC	2p5k	A	64	15

Continued on next page

Table B – *Continued from previous page*

Protein Structure	PDB id	Chain	Number of Residues	Number of Mutable Residues
CusF-Ag(I) residues 10-88 from Escherichia coli	2qcp	X	80	18
DHFR R-67	2rh2	A	62	15
Cytochrome c555 from Aquifex aeolicus	2zxy	A	87	18
Caulobacter crescentus ClpS protease adaptor protein in complex with a N-end rule peptide	3dnj	A	190	18
GB1	3fil	A	112	14
C-terminal domain of the Rous Sarcoma Virus capsid protein	3g21	A	77	15
An Hfq protein from Synechocystis sp.	3hfo	A	210	11
Cold shock protein E from Salmonella typhimurium	3i2z	A	142	19
Arm-type binding domain of HPI integrase	3jtz	A	88	17

Table C. PDB ids, chain, and number of mutable residues for 28 surface design problems.

Protein Structure	PDB id	Chain	Number of Residues	Number of Mutable Residues
Scorpion Toxin protein	1aho	A	64	15
Cytochrome C-553 from B. Pasteurii	1c75	A	71	16
Maize Lipid-Transfer Protein	1fk5	A	93	17
Bovine Pancreatic Trpisin Inhibitor	1g6x	A	58	14
Oxidized [4Fe-4S] Ferredoxin from B. Thermoprotelyticus	1iqz	A	81	18
NapB subunit of the periplasmic nitrate reductase from H. influenzae.	1jni	A	123	17
Ribonuclease from S. Aureofaciens	1lni	A	192	17
TAP UBA Domain and FXFG Nucleoprotein peptide	1oai	A	68	17
syntenin PDZ2	1r6j	A	82	19

Continued on next page

Table C – *Continued from previous page*

Protein Structure	PDB id	Chain	Number of Residues	Number of Mutable Residues
Apo acyl carrier protein from E. coli	1t8k	A	77	17
Type 2 non specific lipid transfer protein from wheat	1tuk	A	67	16
Dissimilatory sulfite reductase D (DsrD)	1ucr	A	156	18
Type III Antifreeze Protein RD1 from an Antarctic Eel Pout	1ucs	A	64	14
Cobrotoxin	1v6p	A	124	13
Dodecin with Flavin	2cc6	A	68	19
S. pneumoniae PhtA histidine triad domain	2cs7	A	165	13
Rubredoxin from Desulfovibrio gigas	2dsx	A	52	16
Complex Between the Pair of the LDL Receptor Ligand-Binding Modules 3-4 and the Receptor Associated Protein (RAP).	2fcw	B	189	19

Continued on next page

Table C – *Continued from previous page*

Protein Structure	PDB id	Chain	Number of Residues	Number of Mutable Residues
Alzheimer's Amyloid Precursor Protein (APP) Copper Binding Domain	2fma	A	59	14
N-terminal Domain of Ribosomal Protein L9 (NTL9) K12M	2hba	A	104	16
N15: an ortholog of lambda Cro	2hin	A	142	19
Extracellular Domain of the Type II BMP Receptor	2hlr	A	100	11
Human CD59	2j8b	A	79	14
The second SH3 domain from ponsin	2o9s	A	67	15
N-terminal domain of AhrC	2p5k	A	64	18
DHFR R-67	2rh2	A	62	14
NusB-S10 transcription antitermination complex.	3d3b	J	87	18
GB1	3fil	A	112	18

Table D. PDB ids, chain, and mutable residues for 6 retrospective design problems.

Protein Structure	PDB id	Chain	Number of Residues	Number of Mutable Residues	Mutable Residues
Protein L	1hz5	A	61	15	3, 4, 6, 7, 8, 9, 10, 14, 16, 18, 20, 21, 23, 25, 26
U1A	1urn	A	64	16	78, 79, 80, 82, 83, 85, 86, 87, 88, 90, 91, 92, 93, 94, 96, 97
Engrailed Homeodomain of D. Melanogaster.	1enh	A	66	12	43, 44, 46, 47, 48, 50, 51, 52, 53, 54, 55, 56
Symmetric protein homodimer	2mg4	A	66	19	8, 11, 14, 15, 16, 18, 19, 20, 22, 23, 24, 44, 47, 48, 51, 52, 54, 55, 56
Acylphosphatase	2acy	A	98	18	5, 6, 8, 10, 11, 12, 13, 14, 16, 74, 76, 77, 78, 79, 81, 83, 84, 85

Continued on next page

Table D – *Continued from previous page*

Protein Structure	PDB id	Chain	Number of Residues	Number of Mutable Residues	Mutable Residues
Designed procarboxypeptidase	1vjq	A	73	26	10, 11, 12, 13, 14, 15, 16, 17, 18, 19, 21, 22, 24, 25, 27, 28, 30, 31, 32, 36, 39, 41, 42, 43, 44, 45

References

1. Shah PS, Hom GK, Ross SA, Lassila JK, Crowhurst KA, Mayo SL. Full-sequence Computational Design and Solution Structure of a Thermostable Protein Variant. Journal of Molecular Biology. 2007;372(1):1–6. Available from: `http://dx.doi.org/10.1016/j.jmb.2007.06.032`.

2. Dantas G, Kuhlman B, Callender D, Wong M, Baker D. A large scale test of computational protein design: folding and stability of nine completely redesigned globular proteins. Journal of molecular biology. 2003;332(2):449–460.

3. Mou Y, Huang PS, Hsu FC, Huang SJ, Mayo SL. Computational design

and experimental verification of a symmetric protein homodimer. Proceedings of the National Academy of Sciences of the United States of America. 2015;112(34):10714–10719. Available from: http://dx.doi.org/10.1073/pnas.1505072112.

4. Dantas G, Corrent C, Reichow SL, Havranek JJ, Eletr ZM, Isern NG, et al. High-resolution Structural and Thermodynamic Analysis of Extreme Stabilization of Human Procarboxypeptidase by Computational Protein Design. Journal of Molecular Biology. 2007;366(4):1209–1221. Available from: http://dx.doi.org/10.1016/j.jmb.2006.11.080.

5. Word JM, Lovell SC, LaBean TH, Taylor HC, Zalis ME, Presley BK, et al. Visualizing and quantifying molecular goodness-of-fit: small-probe contact dots with explicit hydrogen atoms. Journal of molecular biology. 1999 Jan;285(4):1711–1733.

6. Case DA, Darden TA, Cheatham TE, III, Simmerling CL, Wang J, et al.. AMBER 9. University of Calinfornia, San Francisco; 2006.

7. Cornell WD, Cieplak P, Bayly CI, Gould IR, Merz KM, Ferguson DM, et al. A Second Generation Force Field for the Simulation of Proteins, Nucleic Acids, and Organic Molecules. Journal of the American Chemical Society. 1995 May;117(19):5179–5197.

8. Lazaridis T, Karplus M. Effective energy function for proteins in solution. Proteins: Structure, Function, and Bioinformatics. 1999 May;35(2):133–152.

9. Lippow SM, Wittrup KD, Tidor B. Computational design of antibody-affinity improvement beyond in vivo maturation. Nature biotechnology. 2007 Oct;25(10):1171–1176.

10. Lovell SC, Word JM, Richardson JS, Richardson DC. The penultimate rotamer library. Proteins: Structure, Function, and Bioinformatics. 2000;40(3):389–408.
