# Supplementary figures and images for: A critical analysis of computational protein design with sparse residue interaction graphs
Source: PLoS Comput Biol. 2017 Mar 30;13(3):e1005346. doi: 10.1371/journal.pcbi.1005346 (PMC5391103; doi:10.1371/journal.pcbi.1005346)

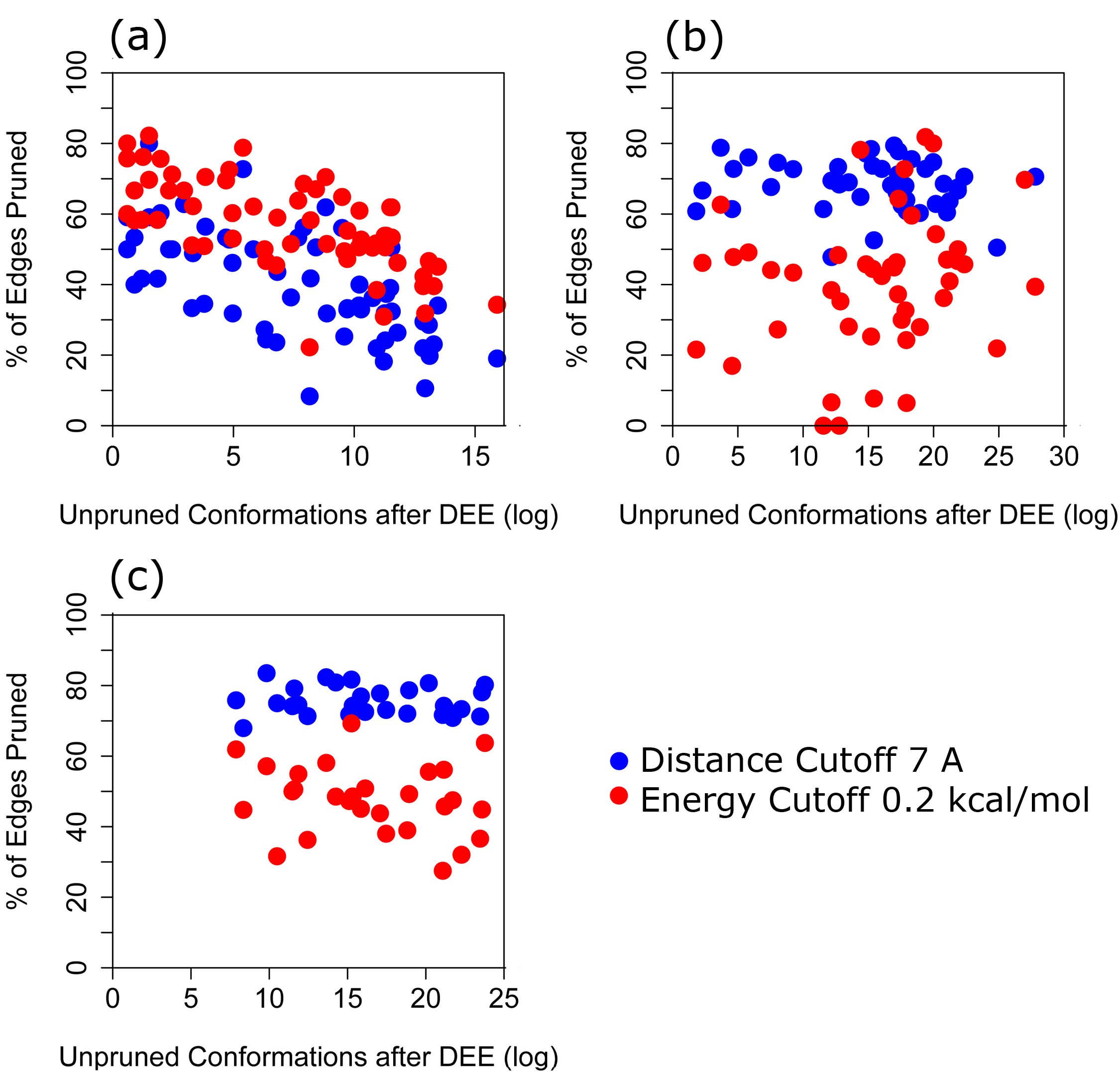

Supplement: S1 Fig — Number of unpruned conformations left after DEE vs. the percentage of edges deleted from the residue interaction graph. Two data points are plotted for each design problem: with distance cutoff δ = 7 Å (blue), and energy cutoff α = 0.2 kcal/mol (red). (a) 62 core design problems, (b) 46 boundary design problems, and (c) 28 surface design problems. (TIF) [file pcbi.1005346.s004.tif]

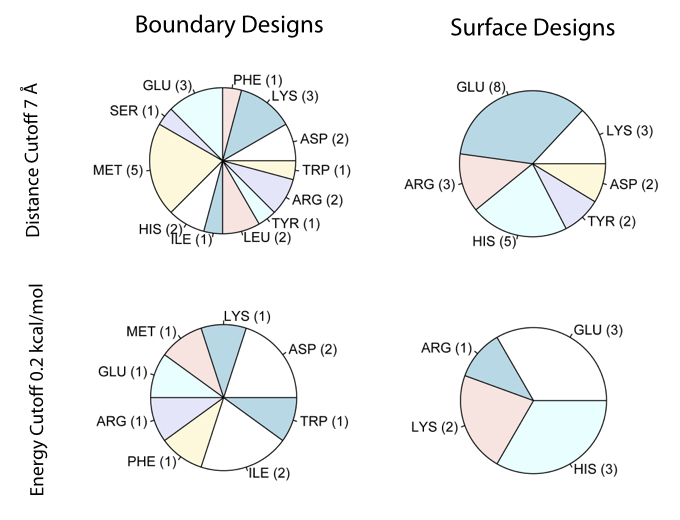

Supplement: S2 Fig — Amino acid identities of residues in the full GMEC which were mutated in the sparse GMEC, for boundary and surface protein design problems with distance cutoff δ = 7 Å and energy cutoff α = 0.2 kcal/mol. The number in parenthesis indicates the cumulative number of residues across all design problems for which that amino acid was different in the sparse GMEC. (TIF) [file pcbi.1005346.s005.tif]
