# Supplementary material for: A critical analysis of computational protein design with sparse residue interaction graphs
Source: PLoS Comput Biol. 2017 Mar 30;13(3):e1005346. doi: 10.1371/journal.pcbi.1005346 (PMC5391103; doi:10.1371/journal.pcbi.1005346)
Supplement: S1 Table — Sequence differences between the full and the sparse GMEC for boundary design problems, for distance cutoff δ = 7 Å and energy cutoff α = 0.2 kcal/mol. A dash (“−”) indicates that the amino acid identity was the same in the full and the sparse GMEC. (PDF) [file pcbi.1005346.s006.pdf]

S1 Table

PDB ID	Residue Number	Amino Acid in Full GMEC	Mutated Amino Acid in Sparse GMEC ($\delta = 7$ Å)	Mutated Amino Acid in Sparse GMEC ($\alpha = 0.2$ kcal/mol)
1IQZ	14	Asp	Lys	Lys
	55	Lys	Arg	Arg
	80	Phe	Lys	–
1PSR	32	Glu	Gln	–
1UCS	3	Ser	Ala	–
	46	Met	Asn	–
	47	Met	Arg	–
1VBW	8	Met	Glu	Glu
2B97	15	His	Lys	–
	18	Ile	Arg	–
	24	Lys	Glu	–
	61	Met	Arg	–
	54	Glu	–	Ile
	55	Arg	–	His
2CS7	7	Asp	Arg	Arg
2DSX	3	Lys	Ile	–
	14	Glu	Arg	–
2ZXY	33	Met	Asp	–
3DNJ	46	His	Arg	–
	110	Phe	–	Asn
3FIL	16	Leu	Met	–
3HFO	37	Ile	–	Arg
3G21	153	Tyr	His	–
	172	Arg	Glu	–
	184	Trp	Asp	–
	188	Leu	Arg	–
	219	Glu	Arg	–
	223	Arg	Met	–
1C75	47	Trp	–	Phe
	75	Ile	–	Met
