# Supplementary material for: A critical analysis of computational protein design with sparse residue interaction graphs
Source: PLoS Comput Biol. 2017 Mar 30;13(3):e1005346. doi: 10.1371/journal.pcbi.1005346 (PMC5391103; doi:10.1371/journal.pcbi.1005346)
Supplement: S2 Table — Sequence differences between the full and the sparse GMEC for surface design problems, for distance cutoff δ = 7 Å and energy cutoff α = 0.2 kcal/mol. A dash (“−”) indicates that the amino acid identity was the same in the full and the sparse GMEC. (PDF) [file pcbi.1005346.s007.pdf]

# S2 Table

| PDB ID | Residue Number | Amino Acid in Full GMEC | Mutated Amino Acid in Sparse GMEC ($\delta = 7$ Å) | Mutated Amino Acid in Sparse GMEC ($\alpha = 0.2$ kcal/mol) |
|---|---|---|---|---|
| 1C75 | 56 | Lys | Glu | – |
| | 59 | Glu | Lys | – |
| 1UCS | 16 | Glu | Arg | Arg |
| | 18 | Arg | Glu | Glu |
| 2CS7 | 17 | Lys | Arg | Arg |
| | 32 | Glu | His | His |
| | 53 | His | Arg | – |
| 2FMA | 161 | Glu | Arg | – |
| 2HLR | 96 | Tyr | Arg | – |
| 2J8B | 15 | Glu | His | – |
| | 20 | Arg | His | – |
| | 24 | Glu | Lys | – |
| | 43 | Asp | Glu | – |
| | 71 | Arg | His | – |
| 2O9S | 838 | His | Arg | Tyr |
| 2RH2 | 23 | His | Arg | – |
| 3FIL | 13 | His | Arg | – |
| | 22 | Asp | His | – |
| | 55 | Lys | Arg | – |
| 1IQZ | 23 | Tyr | Arg | – |
| | 28 | His | Arg | Arg |
| | 45 | Glu | Thr | – |
| | 41 | Lys | – | Arg |
| 1V6P | 6 | Glu | His | His |
| | 56 | His | – | Arg |
