# Supplementary material for: A critical analysis of computational protein design with sparse residue interaction graphs
Source: PLoS Comput Biol. 2017 Mar 30;13(3):e1005346. doi: 10.1371/journal.pcbi.1005346 (PMC5391103; doi:10.1371/journal.pcbi.1005346)
Supplement: S3 Table — The table shows the sequences of the sparse and full GMEC. Residues at which the sparse or full GMEC has the same amino acid identity as the thermostabilized mutant are in bold. Residues at which the sparse or full GMEC has the same amino acid identity as the less stable wild-type are not in bold. (PDF) [file pcbi.1005346.s008.pdf]

S3 Table

Table A. Sequence Correlation Between Designed Mutant and Wild Type. The table shows the sequences of the sparse and full GMEC. Residues at which the sparse or full GMEC has the same amino acid identity as the thermostabilized mutant are in bold. Residues at which the sparse or full GMEC has the same amino acid identity as the less stable wild-type are not in bold.

PDB id		Sequence
2ACY	Sparse GMEC:	AEGDT**Y**I**Q**V**KW**EIFGKVQGVFFRKYTQAEGKKLGLVGWVQNTD<br>QGTVQGQLQGPASKVRHMQEWLETKGSPKS**D**IDR**TV**FHNEK**R**I<br>VKLDYTDFQIVK
	Full GMEC:	AEGDT**Y**ISVD**W**EI**K**G**D**VQGVFFRKYTQAEGKKLGLVGWVQNTD<br>QGTVQGQLQGPASKVRHMQEWLETKGSPKS**D**I**K**R**TV**FHNEK**R**I<br>VKLDYTDFQIVK
1URN	Sparse GMEC:	AVPETRPNHTIYINNLNEKIKKDELKKSLHAIFSRFGQILDIL<br>VSRSLKMRGQAFVIFKEVSSATNALRSMQGFPFYDKPMRI**T**Y**S**<br>KTD**Q**D**Q**IAK**D**
	Full GMEC:	AVPETRPNHTIYINNLNEKIKKDELKKSLHAIFSRFGQILDIL<br>VSRSLKMRGQAFVIFKEVSSATNALRSMQGFPFYDKPMRI**T**Y**S**<br>KTD**Q**DIIAKM
1HZ5	Sparse GMEC:	EVTIK**V**N**F**IFA**D**G**K**TQT**I**EFKGTFEKATSEAYAYADTLKKDNG<br>EWTVDVADKGYTLNIKFAG
	Full GMEC:	E**K**TIK**V**N**F**IFA**D**G**K**TQT**I**EFKGT**E**EKATSEAYAYADTLKKDNG<br>EWTVDVADKGYTLNIKFAG
1ENH	Sparse GMEC:	TAFSSEQLARLKREFNENRYLTERRRQQLSSELGLNE**E**QI**RQ**W<br>F**EE**KR**Q**KI
	Full GMEC:	TAFSSEQLARLKREFNENRYLTERRRQQLSSELGLNE**E**QI**R**IW<br>F**EE**KR**QR**I
2MG4	Sparse GMEC:	MEKRPRT**E**FS**E**EQ**KK**RL**D**REF**Y**E**D**RYLTERRRQQLSSELGLNE<br>**E**QI**ER**WF**RR**K**EQQ**IGWSHPQFEK
	Full GMEC:	MEKRPRT**E**FS**E**EQ**KK**RL**D**REF**Y**E**D**RYLTERRRQQLSSELGLNE<br>**E**QI**ER**WF**R**NKR**QQ**IGWSHPQFEK
